# Supplementary material for: TRIM21‐mediated proteasomal degradation of SAMHD1 regulates its antiviral activity
Source: EMBO Rep. 2019 Dec 4;21(1):e47528. doi: 10.15252/embr.201847528 (PMC6944907; doi:10.15252/embr.201847528)
Supplement: Supplementary file 1 — Appendix [file EMBR-21-e47528-s001.pdf]

Appendix for "TRIM21-mediated proteasomal degradation of SAMHD1 regulates its antiviral activity"

**Appendix Figure S1**	**page 2**
**Appendix Table S1**	**page 3**
**Appendix Table S2**	**page 4**
**Appendix Table S3**	**page 8**

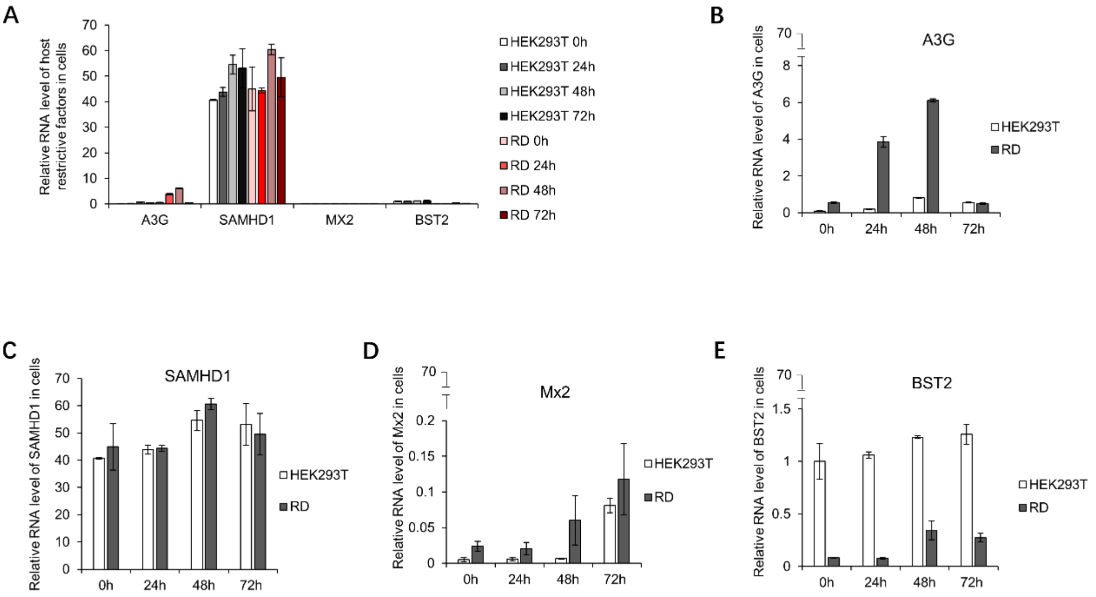

Appendix Figure S1. The mRNA levels of the host restriction factors after EV71 infection.

The mRNA levels of A3G (A), SAMHD1 (B), Mx2 (C), BST-2 (D) were detected by RT-qPCR in infected HEK293T or RD cells at indicated time point post infection. The mRNA levels at 1 h was set as 100%. (E) The mRNA expression levels of the target genes were normalized to GAPDH. (n=3, mean±SD, no significance, * *P* < 0.05, paired t-test).

Appendix Table S1. Proteins binding to SAMHD1 detected by Mass spectrometry.

Protein name	Relative abundance (%)	Number of peptides (Confidence>95%)
Actin	30.8	54
IGK@	13.3	18
Vimentin	1.3	8
Serotransferrin	0.6	7
ADE2	2.8	7
Alpha-1-antitrypsin	1.2	5
**TRIM21**	**0.7**	**4**
Destrin	2.9	4
Tubulin alpha-4A	0.6	3
Tubulin alpha-2A	1.0	3
HSPA8	0.3	3
Nucleolin	0.2	3
Lactotransferrin	0.4	3
PPIA	2.2	2
RCN2	0.6	2
ZG16B	1.6	2
Complement C3	0.1	2
EF1G	0.7	2
NRK	0.3	2
OAT	0.5	2
GON4L	0.1	2
DNPEP	0.3	2

Appendix Table S2. Primers for plasmid construction.

Primer	Sequence	Enzyme site	Purpose
SAMHD1-F	GCGTCGACATGCAGCGA GCCGATTCC	SalI	SAMHD1 -VR1012
SAMHD1-HA-R	CGGGATCCTCAAGCGTA ATCTGGAACATCGTATG GGTACATTGGGTCATCT TTAAAAAGC	BamHI	SAMHD1-HA -VR1012
SAMHD1-flag-R	CGGGATCCTCACTTATC GTCGTCATCCTTGTAAT CCATTGGGTCATCTTTAA AAAGC	BamHI	SAMHD1-flag-VR1012
SAMHD1 109-626 F	GCGTCGACATGCAAATC CACGTTGATACAATG	SalI	SAMHD1 109-626-VR1012
SAMHD1 120-626 F	GCGTCGACATGGATCCT ATCCATGGCCAC	SalI	SAMHD1 120-626-VR1012
SAMHD1 1-547-HA-R	CGGGATCCTCACGCGTA ATCTGGGACGTCGTAAG GGTACTCTGCAAATTTCT CTGGC	BamHI	SAMHD1 1-547-HA-VR1012
SAMHD1 1-547-flag-R	CGGGATCCTCACTTGTC ATCGTCGTCCTTGTAGT CCTCTGCAAATTTCTCTG GC	BamHI	SAMHD1 1-547-flag-VR1012
SAMHD1 Q548A-F	CAGAGAAATTTGCAGAG GCGCTGATTCGAGTATA TTG	None	SAMHD1-Q548A-VR1012
SAMHD1 Q548A-R	CAATATACTCGAATCAG CGCCTCTGCAAATTTCT CTG	None	SAMHD1-Q548A-VR1012
SAMHD1-T592D-F	GGTGAGCCCCTAATTCA GAGACATACAGGTTC	None	SAMHD1-T592D-VR1012
SAMHD1-T592D-R	GCGTCGACATGTACCCT TACGACGTCCCAGATTA CGCGATTATTAGGGGCT CACCAGG	None	SAMHD1-T592D-VR1012
SAMHD1-K555R-F	GATTCGAGTATATTGTA GGAAGGTGGACAGAAA G	None	SAMHD1-K555R-VR1012
SAMHD1-K555R-R	CTACAATATACTCGAATC AGCTGCTCTG	None	SAMHD1-K555R-VR1012
SAMHD1-K556R-F	TCGAGTATATTGTAAGA GGGTGGACAGAAAGAG	None	SAMHD1-K556R-VR1012
SAMHD1-K556R-R	CTCTTACAATATACTCGA	None	SAMHD1-K556R-VR1012

	ATCAGCTGC		
SAMHD1-K560R-F	AGAAGGTGGACAGAAG GAGTTTGTATGCCGC	None	SAMHD1-K560R-VR1012
SAMHD1-K560R-R	CTTCTGTCCACCTTCTTA CAATATAC	None	SAMHD1-K560R-VR1012
SAMHD1-K580R-F	GACAGAAATTTCACCAG GCCGCAGGATGGC	None	SAMHD1-K580R-VR1012
SAMHD1-K580R-R	GACAGAAATTTCACCAG GCCGCAGGATGGC	None	SAMHD1-K580R-VR1012
SAMHD1-K595R-F	CTCATAACACCTCAAAG AAAGGAATGGAACGAC	None	SAMHD1-K595R-VR1012
SAMHD1-K595R-R	CTTTGAGGTGTTATGAG TGGGGCTAT	None	SAMHD1-K595R-VR1012
SAMHD1-K596R-F	CATAACACCTCAAAAAA GGGAATGGAACGACAG	None	SAMHD1-K596R-VR1012
SAMHD1-K596R-R	CTTTTTTGAGGTGTTATG AGTGGGGCTA	None	SAMHD1-K596R-VR1012
SAMHD1-K615R-F	CCGAGAAGCATCCAGAA GCAGAGTCCAGC	None	SAMHD1-K615R-VR1012
SAMHD1-K615R-R	CTGGATGCTTCTCGGAG GCGAGTTG	None	SAMHD1-K615R-VR1012
SAMHD1-K622R-F	AGAGTCCAGCTTTTTAG AGATGACCCAATGTAC	None	SAMHD1-K622R-VR1012
SAMHD1-K622R-R	GTACATTGGGTCATCTC TAAAAAGCTGGACTCT	None	SAMHD1-K622R-VR1012
SAMHD1-T114A-F	CAAATCCACGTTGATGC AATGAAGGTAATTAATG	None	SAMHD1-T114A-VR1012
SAMHD1-T114A-R	CATCAACGTGGATTTGA ACCAATCG	None	SAMHD1-T114A-VR1012
SAMHD1-G153S-F	AACAGCTGGGAGGTAGT TACTATGTTTTTCCA	None	SAMHD1-G153S-VR1012
SAMHD1-G153S-R	TACCTCCCAGCTGTTTG ATGTATC	None	SAMHD1-G153S-VR1012
SAMHD1-G183R-F	TAGTTCACGCACTGAGA GAAAAACAACCAGAGC	None	SAMHD1-G183R-VR1012
SAMHD1-G183R-R	GAGCAGTGCGTGAACTA GACATCCTG	None	SAMHD1-G183R-VR1012
SAMHD1-N535D-F	CAATCAGGATTACTAAA GACCAGGTTTCACAAC	None	SAMHD1-N535D-VR1012
SAMHD1-N535D-R	CTTTAGTAATCCTGATTG CTCTGTTGG	None	SAMHD1-N535D-VR1012
TRIM21-F	GCGTCGACATGGCTTCA GCAGCACGC	SalI	TRIM21-VR1012

TRIM21-HA-R	GAAGATCTTCACGCGTAATCTGGGACGTCGTAAGGGTAATAGTCAGTGGATCCTTGTG	BglII	TRIM21-HA-VR1012
TRIM21-flag-R	GAAGATCTTCACTTGTCATCGTCGTCCTTGTAGTCATAGTCAGTGGATCCTTGTG	BglII	TRIM21-flag-VR1012
TRIM21-△RING-F	GCGTCGACATGCCTGTGTGCCGGCAGCG	SalI	TRIM21-△RING-VR1012
TRIM21-△B box-F	AGCGTCTGTCCTGTGCTGAGAAGAAAGCAGG	None	TRIM21-△B box-VR1012
TRIM21-△B box-F	GCACAGGACAGACGCTGCCCCCACC	None	TRIM21-△B box-VR1012
TRIM21-△PRY-R	TCCAGAACTCAGGAGTCAGCTCTTTCACTCTG	None	TRIM21-△PRY-VR1012
TRIM21-△PRY-R	GACTCCTGAGTTCTGGAGAGGTAATA	None	TRIM21-△PRY-VR1012
TRIM21-C16A-F	TGGGAGGAGGTCACAGCCCCTATCTGCCTGGA	None	TRIM21-C16A-VR1012
TRIM21-C16A-F	GCTGTGACCTCCTCCCACATCATTGT	None	TRIM21-C16A-VR1012
TRIM21-PRYSPRY-F	CGGAATTCGTGTGCCATGTGCCAGGG	EcoRI	TRIM21-PRYSPRY-pGEX-6P-1
TRIM21-PRYSPRY-R	CCGCTCGAGTCAATAGTCAGTGGATCCTTG	XhoI	TRIM21-PRYSPRY-pGEX-6P-1
SAMHD1-sh-F	CCGGAATGTACACGCATGCTGAAGCCTCGAGGCTTCAGCATGCGTGTACATTTTTTTG	None	SAMHD1-sh-pLKO.1
SAMHD1-sh-R	AATTCAAAAAAATGTACACGCATGCTGAAGCCTCGAGGCTTCAGCATGCGTGTACATT	None	SAMHD1-sh-pLKO.1
TRIM21-sh-F1	CCGGAATGCATCTCTCAGGTTGGGACTCGAGTCCCAACCTGAGAGATGCATTTTTTTG	None	TRIM21-sh-pLKO.1
TRIM21-sh-R1	AATTCAAAAAAATGCATCTCTCAGGTTGGGACTCGAGTCCCAACCTGAGAGATGCATT	None	TRIM21-sh-pLKO.1
TRIM21-sh-F2	CCGGAATCTAGGATTCACGCAGAGTCTCGAGACT	None	TRIM21-sh-pLKO.1

	CTGCGTGAATCCTAGATTTTTTTG		
TRIM21-sh-R2	AATTCAAAAAAATCTAGGATTCACGCAGAGTCTCGAGACTCTGCGTGAATCCTAGATT	None	TRIM21-sh-pLKO.1
TRIM21-sh-F3	CCGGAAGAGTGGCTTCTGGACAATTCTCGAGAATTGTCCAGAAGCCACTCTTTTTTTG	None	TRIM21-sh-pLKO.1
TRIM21-sh-R3	AATTCAAAAAAAGAGTGGCTTCTGGACAATTCTCGAGAATTGTCCAGAAGCCACTCTT	None	TRIM21-sh-pLKO.1
IFNAR1-sh-F	CCGGAAGAACTACAGCAGGACTTTGCTCGAGCAAAGTCCTGCTGTAGTTCTTTTTTTG	None	IFNAR1-sh-pLKO.1
IFNAR1-sh-R	AATTCAAAAAAAGAACTACAGCAGGACTTTGCTCGAGCAAAGTCCTGCTGTAGTTCTT	None	IFNAR1-sh-pLKO.1
TRIM21-gRNA-F	AAAGGACGAAACACCTCATCTCAGAGCTAGATCGAGTTTTAGAGCTAGAAATAG	None	CRISPR-TRIM21
TRIM21-gRNA-R	AGGTGTTTCGTCCTTTCCACAAGAT	None	CRISPR-TRIM21
Flag-ubiquitin-F1	TCGACATGGACTACAAGGACGACGATGACAAGATGCAGATCTTCGTCAGAAC	SalI	Flag-ubiquitin-K48-VR1012/ Flag-ubiquitin-K63-VR1012
Flag-ubiquitin-F2	CATGGACTACAAGGACGACGATGACAAGATGCAGATCTTCGTCAGAAC	SalI	Flag-ubiquitin-K48-VR1012/ Flag-ubiquitin-K63-VR1012
Flag-ubiquitin-R1	CTCAACCACCTCTTAGTCTTAAG	BamHI	Flag-ubiquitin-K48-VR1012/ Flag-ubiquitin-K63-VR1012
Flag-ubiquitin-R2	GATCCTCAACCACCTCTTAGTCTTAAG	BamHI	Flag-ubiquitin-K48-VR1012/ Flag-ubiquitin-K63-VR1012

Appendix Table S2. Primers for RT-qPCR.

Primer	Sequence	Purpose
EV71-RT-F	CTTTGTGCGCCTGTTTTATAC	Detect EV71 by RT-qPCR
EV71-RT-R	GGAAACAGAAGTGCTTGATCA	Detect EV71 by RT-qPCR
A3A-RT-F	GGAAGGCATAAGACCTACC	Detect homo-A3A by RT-qPCR
A3A-RT-R	CCGTAAAAGCCACAGAGAAG	Detect homo-A3A by RT-qPCR
A3B-RT-F	CTCCTTTGGGACACAGGG	Detect homo-A3B by RT-qPCR
A3B-RT-R	CCAGGATACAAACCAGGTG	Detect homo-A3B by RT-qPCR
A3C-RT-F	CCAACGATCGGAACGAAAC	Detect homo-A3C by RT-qPCR
A3C-RT-R	GTCGTCGCAGAACCAAGAG	Detect homo-A3C by RT-qPCR
A3D-RT-F	CTCCTTTGGGACACAGGG	Detect homo-A3D by RT-qPCR
A3D-RT-R	CACAGAACCAAGATAAGAAGC	Detect homo-A3D by RT-qPCR
A3F-RT-F	GTGCTTCCTCTCTTGGTTC	Detect homo-A3F by RT-qPCR
A3F-RT-R	GGTGACATTGGGGTGCTC	Detect homo-A3F by RT-qPCR
A3G-RT-F	GATTCTCAGACACTCGATGGATC	Detect homo-A3G by RT-qPCR
A3G-RT-R	CTGGTTCAGCAGGACCCAG	Detect homo-A3G by RT-qPCR
A3H-RT-F	CCGAAACATTCCGCTTACAG	Detect homo-A3H by RT-qPCR
A3H-RT-R	TCAAAGTAGCCTCTCGTGG	Detect homo-A3H by RT-qPCR
SERINC5-RT-F	CGTCCTCTGCTGCATCATG	Detect homo-SERINC5 by RT-qPCR
SERINC5-RT-R	TCTATACACGGCAGAATATCC	Detect homo-SERINC5 by RT-qPCR
BST2-RT-F	GGATAGGAATTCTGGTGCTC	Detect homo-BST2 by RT-qPCR
BST2-RT-R	GGAGATGGGTGACATTGCG	Detect homo-BST2 by RT-qPCR
SAMHD1-RT-F	GGTCCGGAGCAGGTGTG	Detect homo-SAMHD1 by RT-qPCR
SAMHD1-RT-R	CAAAACGAGACTCATCAAGAC	Detect homo-SAMHD1 by RT-qPCR
Mx2-RT-F	AACAATCAGCCACCACCAG	Detect homo-Mx2 by RT-qPCR
Mx2-RT-R	TCCACACCCAGAGCCCG	Detect homo-Mx2 by RT-qPCR
GBP5-RT-F	CGCACTGGCAAATCCTACC	Detect homo-GBP5 by RT-qPCR
GBP5-RT-R	GAACTAATGTGTGATTTGGCC	Detect homo-GBP5 by RT-qPCR
TRIM21-RT-F	AGCTAGCCAACATGGTGAAC	Detect homo-TRIM21 by RT-qPCR
TRIM21-RT-R	TGTTTCCGAGACTGGGCAC	Detect homo-TRIM21 by RT-qPCR
GAPDH-RT-F	CCCATCACCATCTTCCAGG	Detect homo-GAPDH by RT-qPCR
GAPDH-RT-R	TTCTCCATGGTGGTGAAGAC	Detect homo-GAPDH by RT-qPCR
IFNα-RT-F	TGCCTCAAACCCACAGCC	Detect homo-IFNα by RT-qPCR
IFNα-RT-R	CCTTTTGGAACTGGTTGCC	Detect homo-IFNα by RT-qPCR
IFNβ-RT-F	GGATTCCTACAAAGAAGCAG	Detect homo-IFNβ by RT-qPCR
IFNβ-RT-R	CCTTCTGGAACTGCTGCAG	Detect homo-IFNβ by RT-qPCR
IFNAR1-RT-F	TCCAGAAGTACATTTAGAAGC	Detect homo-IFNAR1 by RT-qPCR
IFNAR1-RT-R	CTAACCTGAAGAGAGTTTTTCC	Detect homo-IFNAR1 by RT-qPCR
mus-SAMHD1-RT-F	GATGAGGATCGTCTGGAAG	Detect mus-SAMHD1 by RT-qPCR
mus-SAMHD1-RT-R	GAGGGTGGAACTCAATGTG	Detect mus-SAMHD1 by RT-qPCR
mus-TRIM21-RT-F	GCATGGAGAGAAGCTTCAC	Detect mus-TRIM21 by RT-qPCR
mus-TRIM21-RT-R	TGGATCTTCTCCTGGTATAC	Detect mus-TRIM21 by RT-qPCR
mus-GAPDH-RT-F	CAAGGCCGAGAATGGGAAG	Detect mus-GAPDH by RT-qPCR

mus-GAPDH-RT-R	TCCATGGTGGTGAAGACAC	Detect mus-GAPDH by RT-qPCR
mus-IFNα-RT-F	AGAAGGCTCAAGCCATCCC	Detect mus-IFNα by RT-qPCR
mus-IFNα-RT-R	GTCATTGAGCTGCTGGTGG	Detect mus-IFNα by RT-qPCR
mus-IFNβ-RT-F	GAAAGATCAACCTCACCTAC	Detect mus-IFNβ by RT-qPCR
mus-IFNβ-RT-R	CAGTGCTGGAGAAATTGTTTC	Detect mus-IFNβ by RT-qPCR
