# Supplementary material for: TRIM21‐mediated proteasomal degradation of SAMHD1 regulates its antiviral activity
Source: EMBO Rep. 2019 Dec 4;21(1):e47528. doi: 10.15252/embr.201847528 (PMC6944907; doi:10.15252/embr.201847528)
Supplement: Supplementary file 2 — Expanded View Figures PDF [file EMBR-21-e47528-s002.pdf]

Expanded View Figures

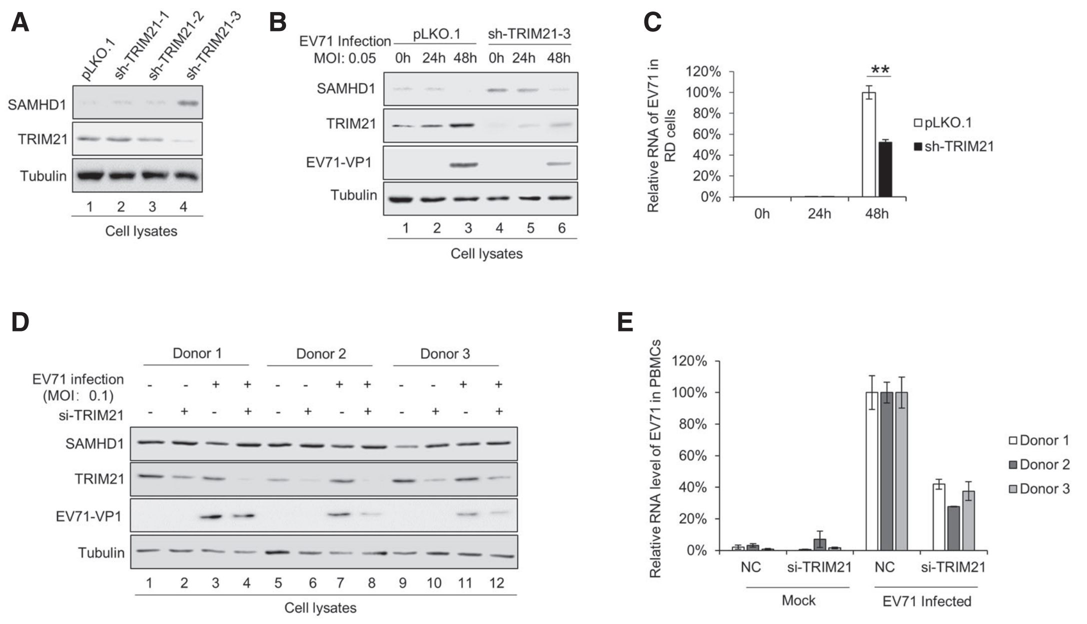

Figure EV1. TRIM21 knockdown releases SAMHD1 restriction on EV71 replication.

A IB analysis of RD cells treated with scrambled shRNA or with TRIM21-specific shRNA with tubulin as a loading control.

B RD-pLKO.1 or RD-shTRIM21-3 cells were infected with EV71 at a MOI of 0.05 for the indicated time and harvested for SAMHD1, TRIM21, and EV71-VP1 detection by IB. Tubulin served as a loading control.

C EV71 RNA levels in (B) were detected by RT–qPCR with GAPDH as a control ($n = 3$, mean ± SD, $^{**}P < 0.01$, paired t-test).

D PBMCs were isolated from three healthy donors and infected with EV71 at 0.1 MOI. After 72 h, PBMCs were harvested and subjected to IB for SAMHD1, TRIM21, and EV71-VP1 detection with tubulin as a control.

E Viral mRNA level of EV71 was detected in PBMCs by RT–qPCR with GAPDH as a control ($n = 3$, mean ± SD).

Source data are available online for this figure.

© 2019 The Authors

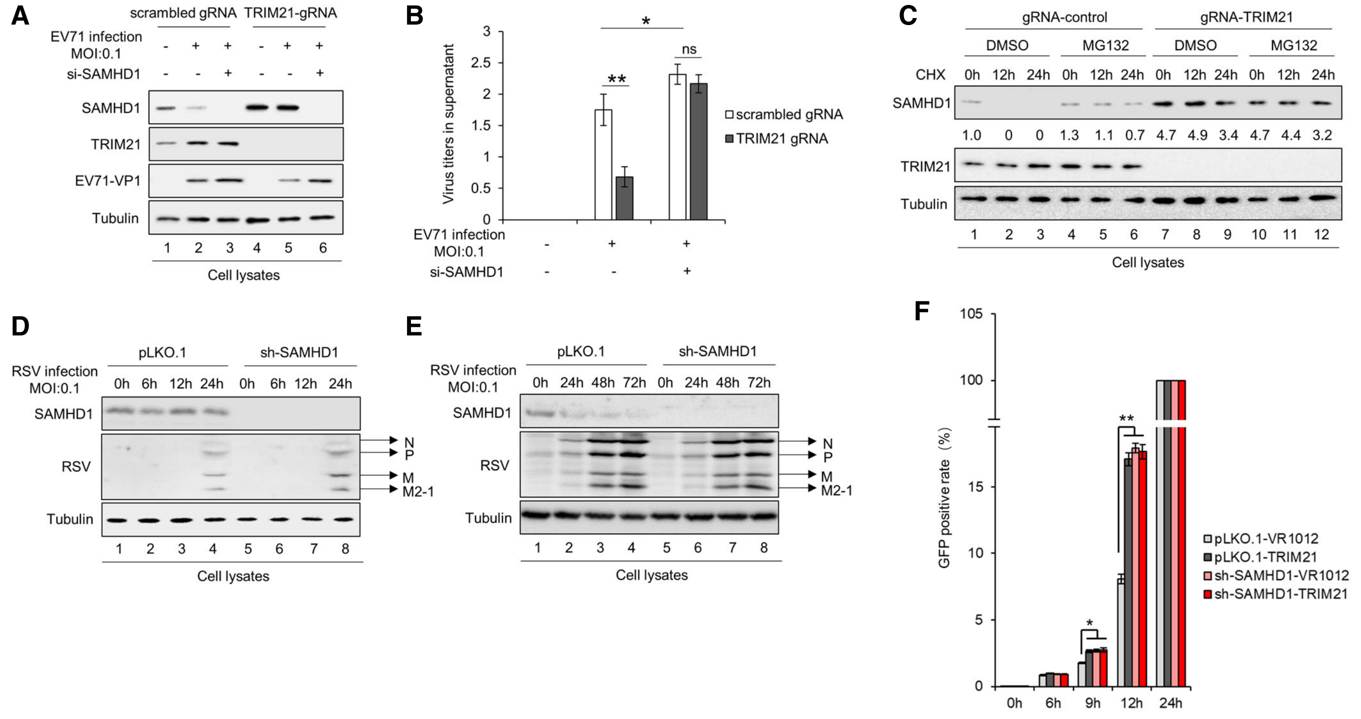

Figure EV2. TRIM21 regulates EV71 replication in SAMHD1-dependent manner.

A, B Stable cell lines scrambled gRNA or TRIM21 gRNA constructed in RD cells were transfected with siRNA-NC or siRNA-SAMHD1, and then infected with EV71 at 0.1 MOI. After 48 h, cells and supernatants were harvested and analysis by IB (A) and titer detection (B) Viral titers in the supernatants were measured by the cytopathic effect method. The results represent the means ± SD from three independent experiments. Statistical significance was analyzed using Student's *t*-test (*$P < 0.05$, **$P < 0.01$).

C Stable cell lines scrambled gRNA or TRIM21 gRNA constructed in RD cells were treated with 100 μg/ml cycloheximide (CHX) and DMSO or 10 μM MG132. Cells were harvested at the indicated time points and then analyzed by IB. The densities of bands were analyzed with ImageJ software to calculate the values relative to that for tubulin.

D, E Stable cell lines pLKO.1 or sh-SAMHD1 constructed in HEK293T cells were infected with RSV at a MOI of 0.1, and then, the cells were harvested at the indicated time points. IB analysis of RSV proteins and SAMHD1 was performed with tubulin as a loading control. (F) SAMHD1 inhibits VSV within 24 h. VR1012 or TRIM21 was transfected into pLKO.1 or sh-SAMHD1 HEK293T cells for 24 h and then infected with VSV-GFP at 0.01 MOI. At the indicated time points, the infectivity of VSV was measured by enumerating GFP-positive cells. (n = 3, mean ± SD, *$P < 0.05$, **$P < 0.01$, paired *t*-test).

Source data are available online for this figure.

 © 2019 The Authors

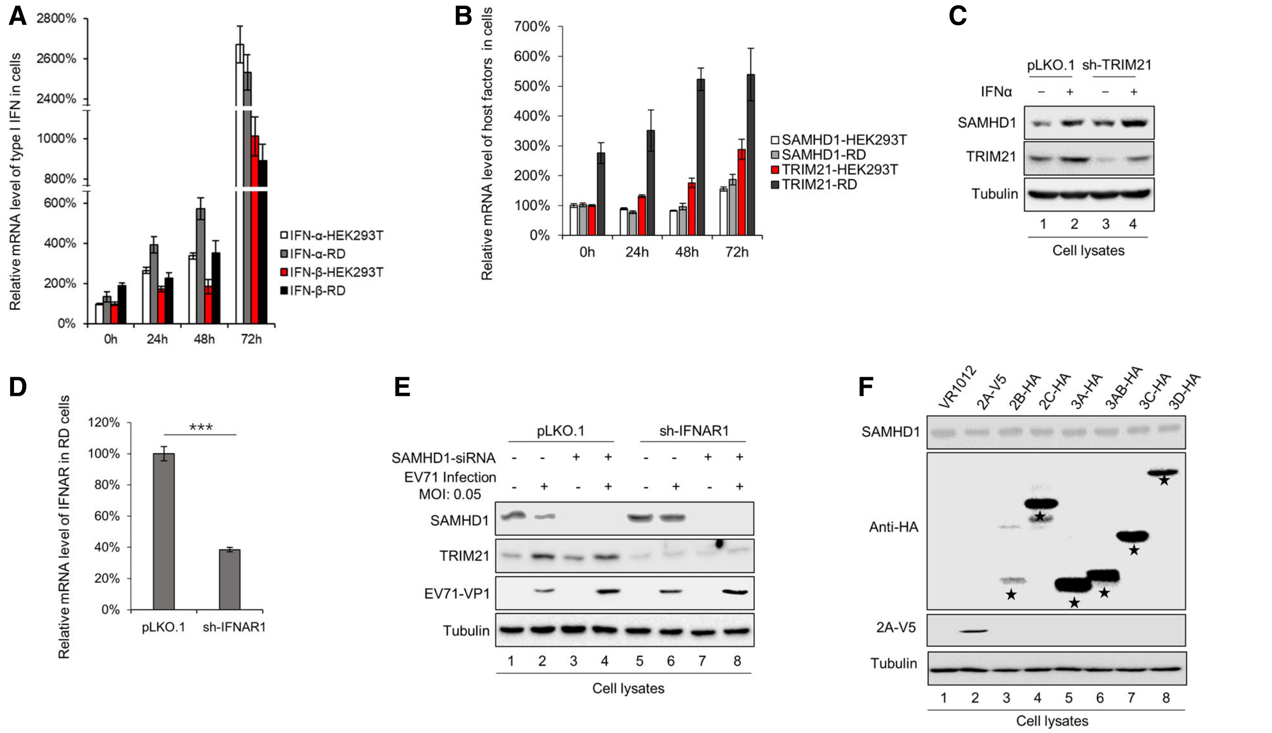

Figure EV3. EV71 upregulates TRIM21 by IFN pathway.

A, B mRNA level of IFN-α and IFN-β as well as SAMHD1 and TRIM21 was upregulated upon EV71 infection. HEK293T and RD cells were infected with EV71 at 0.05 MOI and harvested at different time, and then, mRNA level of IFN-α, IFN-β (A), TRIM21, and SAMHD1 (B) was detected by RT–qPCR with GAPDH as a control (n = 3, mean ± SD).

C TRIM21 is IFN-induced protein. THP1-pLKO.1 or THP1-shTRIM21 cells were stimulated with IFN-α (100 U/ml) for 24 h, and then, SAMHD1 and TRIM21 were detected by Western blot with tubulin as a control.

D, E Upregulation of TRIM21 by EV71 infection is IFNAR1-dependent. (D) IFNAR knockdown was confirmed by RT–qPCR detection (n = 3, mean ± SD, ***P < 0.001, paired t-test). (E) RD-pLKO.1 and RD-shIFNAR cells were transfected with SAMHD1 siRNA as indicated, then infected with EV71 at 0.05 MOI, and harvested for SAMHD1, TRIM21, and EV71-VP1 detection 72 h post-infection by IB with tubulin as a control.

F EV71 nonstructural proteins have no effect on SAMHD1 expression. HEK293T cells were transfected with SAMHD1 plus VR1012 or the indicated EV71 nonstructural proteins for 48 h and subjected to IB analysis with tubulin as a loading control; the blots of EV71 nonstructural proteins were asterisked.

Source data are available online for this figure.

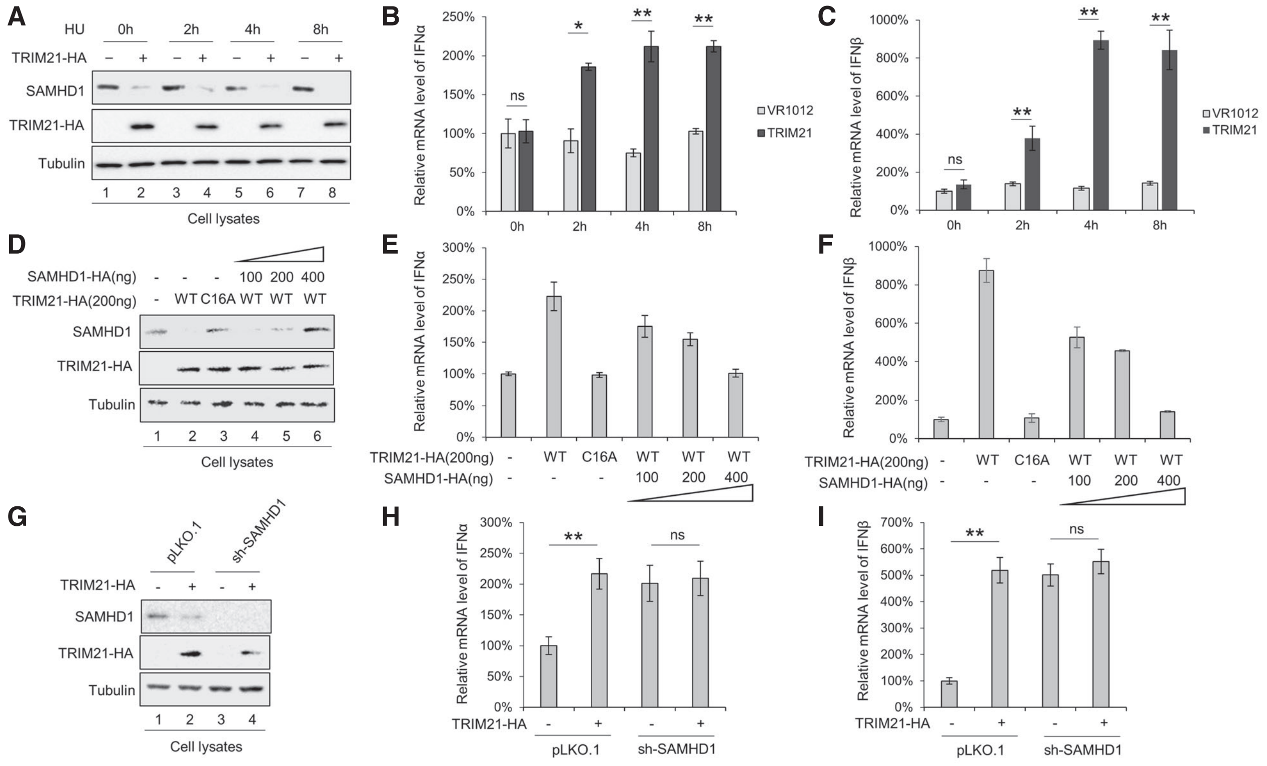

Figure EV4. TRIM21 regulates SAMHD1 function in innate immunity.

A–C TRIM21 enhanced the expression of IFN-α and IFN-β. TRIM21 was transfected into HEK293T cells for 48 h and then treated with hydroxyurea (HU) for the indicated time points. (A) IB analysis of SAMHD1. mRNA levels of IFN-α (B) and IFN-β (C) after treatment with HU (n = 3, mean ± SD, *P < 0.05, **P < 0.01, paired t-test).

D–F Increasing SAMHD1 reduced the TRIM21-induced increase in of IFN-α and IFN-β mRNA. HEK293T cells were cotransfected with TRIM21 WT or C16A mutant and increasing doses of SAMHD1 for 48 h and then treated with HU for 4 h. Cells were subjected to IB analysis (D), and the levels of IFN-α (E) and IFN-β (F) mRNA were detected by RT–qPCR with GAPDH as a control (n = 3, mean ± SD).

G–I TRIM21 enhanced IFN-α and IFN-β production in a SAMHD1-dependent manner. TRIM21 was transfected into pLKO.1 or sh-SAMHD1 HEK293T cells for 48 h, and then, the cells were treated with HU for 4 h. Cells were subjected to IB analysis (G), and the levels of IFN-α (H) and IFN-β (I) mRNA were detected by RT–qPCR with GAPDH as a control (n = 3, mean ± SD, **P < 0.01, paired t-test).

Source data are available online for this figure.

 © 2019 The Authors

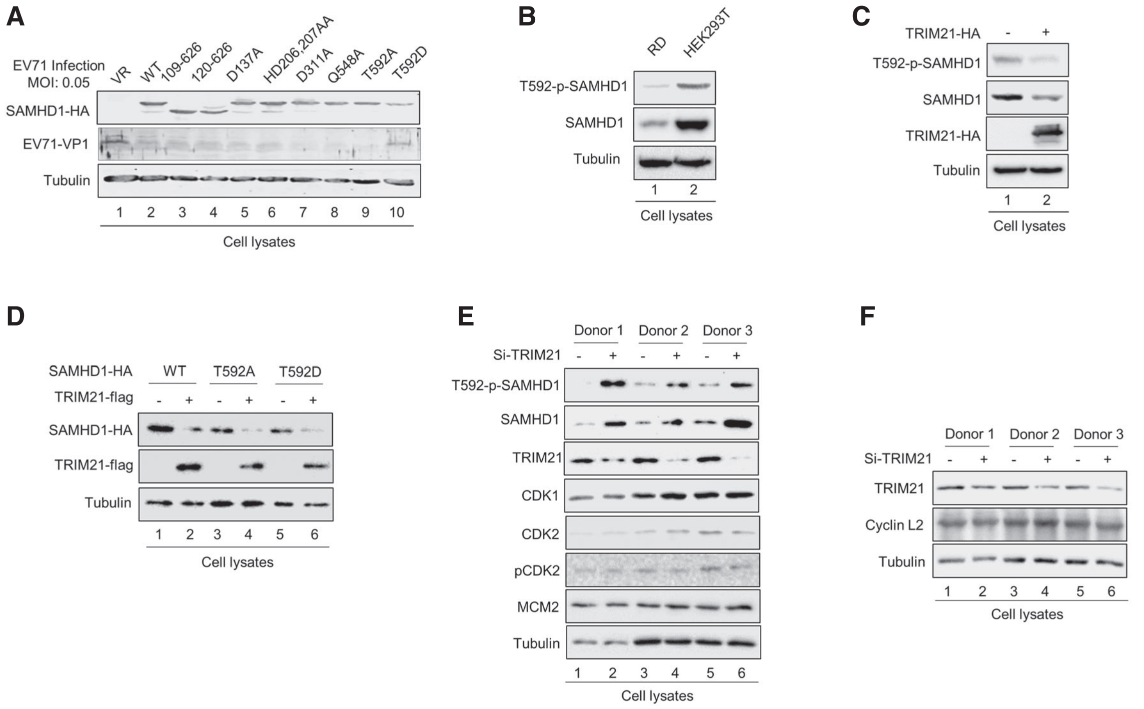

Figure EV5. Phosphorylated SAMHD1 loses the ability to inhibit EV71 replication.

A Stable sh-SAMHD1 HEK293T cells were transfected with VR1012, SAMHD1 WT, or the indicated mutant for 24 h and then infected with EV71 at a MOI of 0.05 for 72 h. Cells were harvested and subjected to IB analysis.

B IB analysis of T592-phosphorylated SAMHD1 and total SAMHD1 in RD and HEK293T cells.

C The effect of TRIM21 on endogenous phosphorylated SAMHD1 and total SAMHD1.

D The effect of TRIM21 on ectopic phosphorylated and unphosphorylated SAMHD1.

E, F TRIM21 directly induced the degradation of SAMHD1 but not via changing cell cycle or upregulating cyclin L2 expression. (E) TRIM21 has no effect on the G0–G1 transition in MDM. (F) TRIM21 has no effect on the expression of cyclin L2 in MDM.

Data information: (A–F) Tubulin served as a loading control.
Source data are available online for this figure.

© 2019 The Authors
