# Supplementary figures and images for: TRIM21‐mediated proteasomal degradation of SAMHD1 regulates its antiviral activity
Source: EMBO Rep. 2019 Dec 4;21(1):e47528. doi: 10.15252/embr.201847528 (PMC6944907; doi:10.15252/embr.201847528)

A

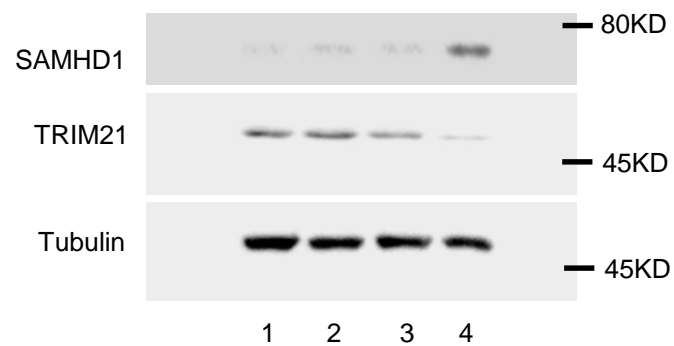

B

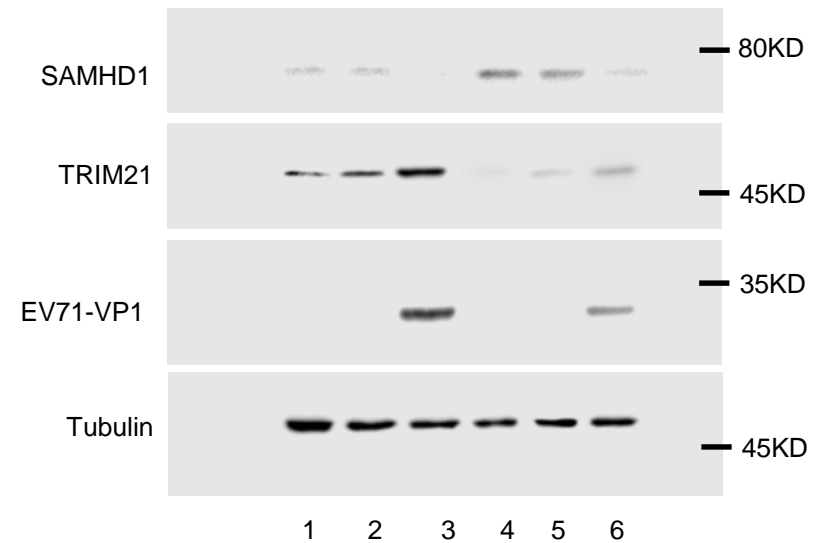

D

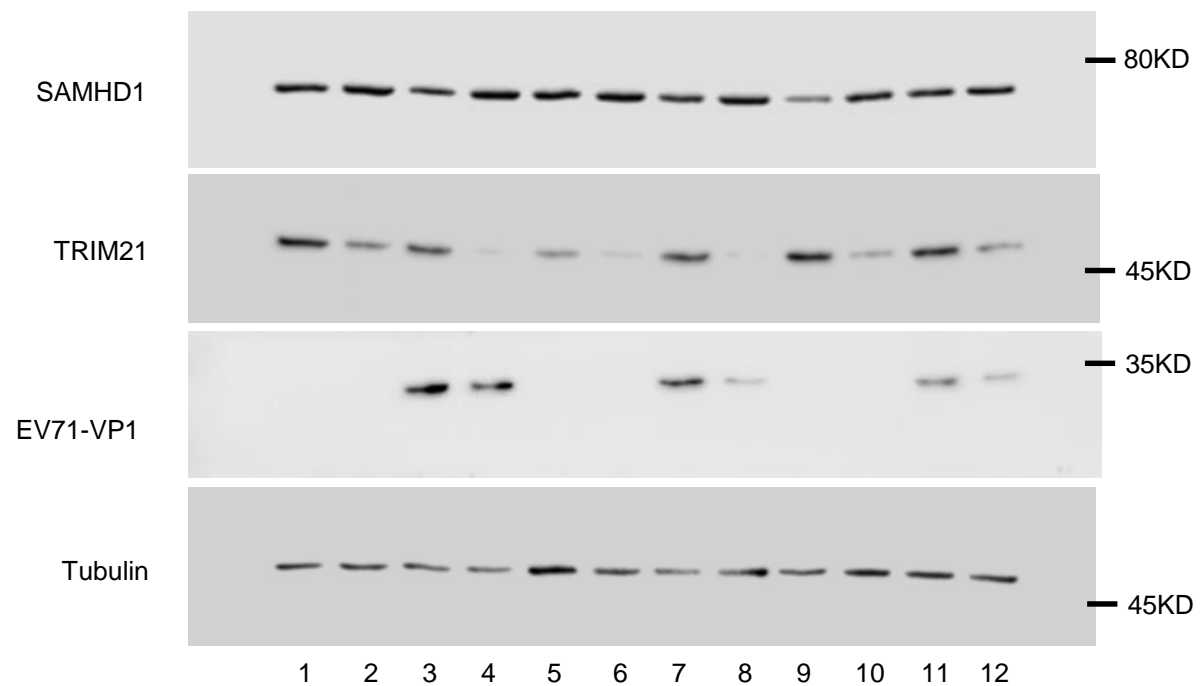

Fig. EV1 source data

Supplement: Supplementary file 3 — Source Data for Expanded View [file EMBR-21-e47528-s010.zip › Source_Data_for_EV_Figures/Fig_EV1/Source_Data_for_FigEV1.pdf]

A

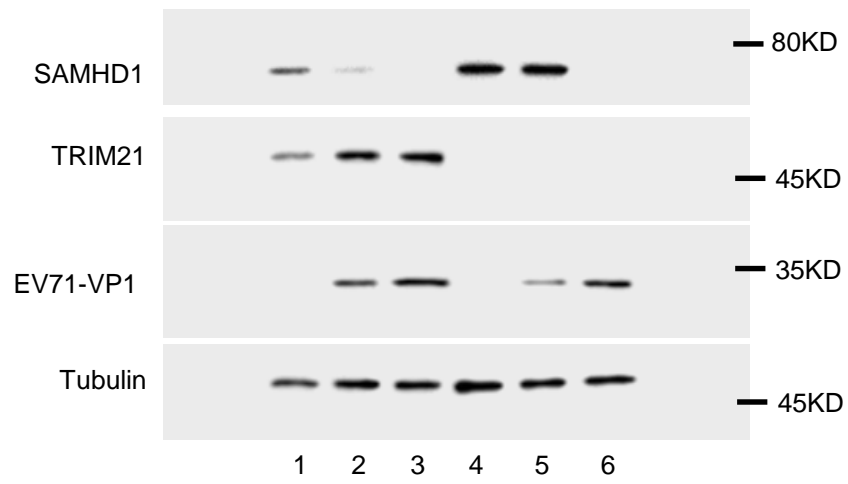

C

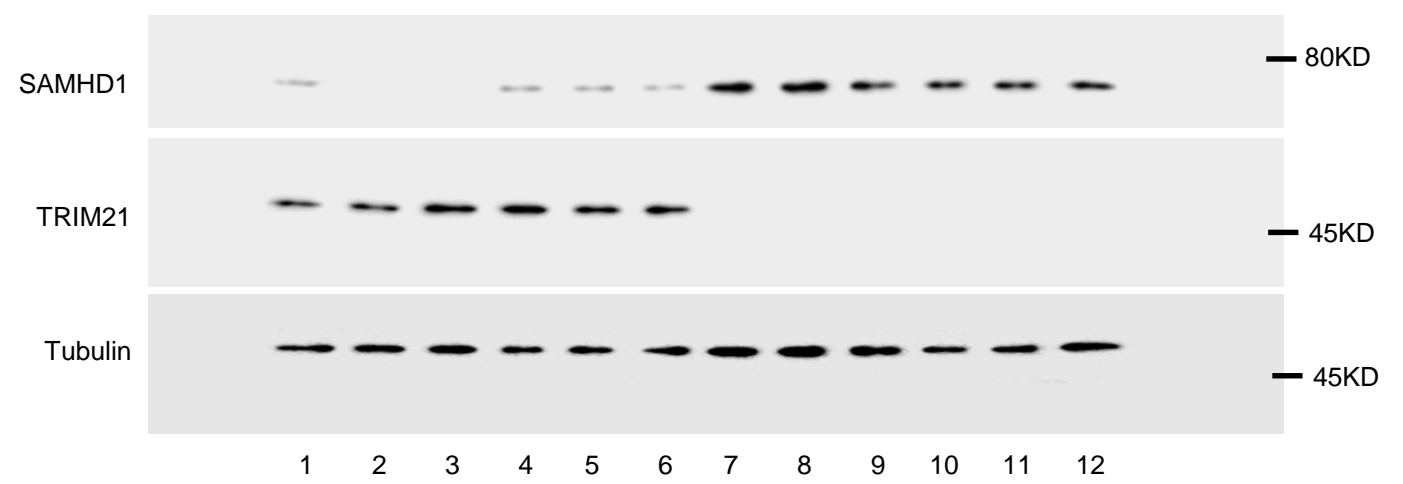

D

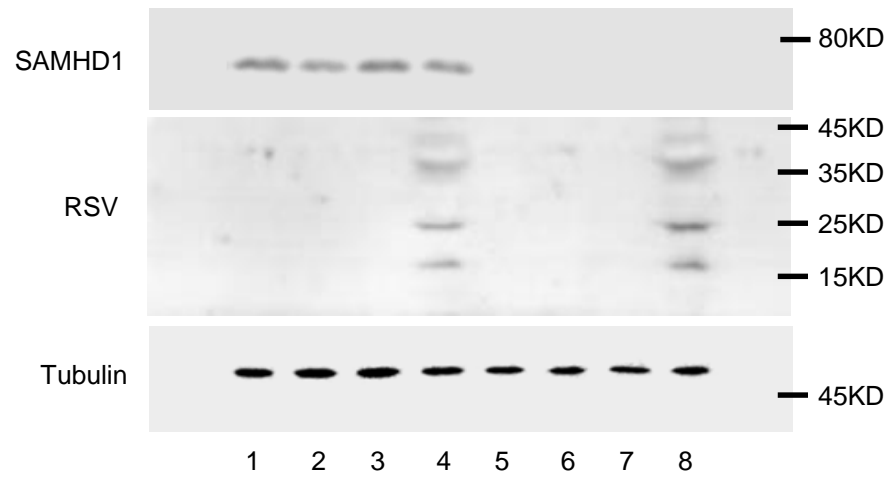

E

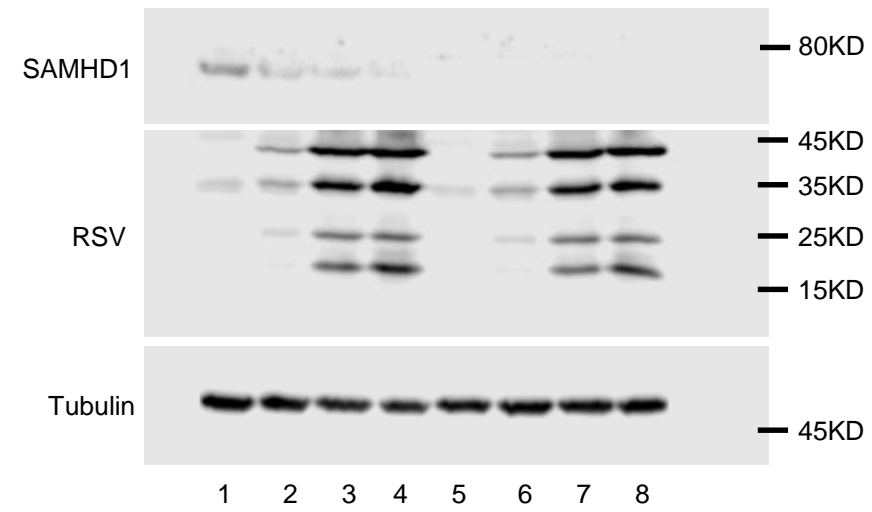

Fig. EV2 source data

Supplement: Supplementary file 3 — Source Data for Expanded View [file EMBR-21-e47528-s010.zip › Source_Data_for_EV_Figures/Fig_EV2/Source_Data_for_FigEV2.pdf]

C

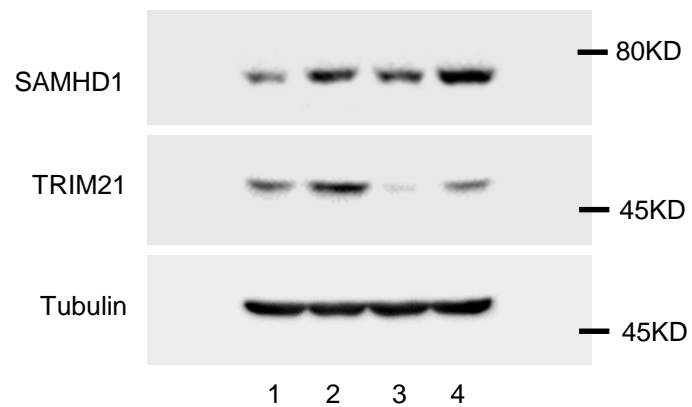

E

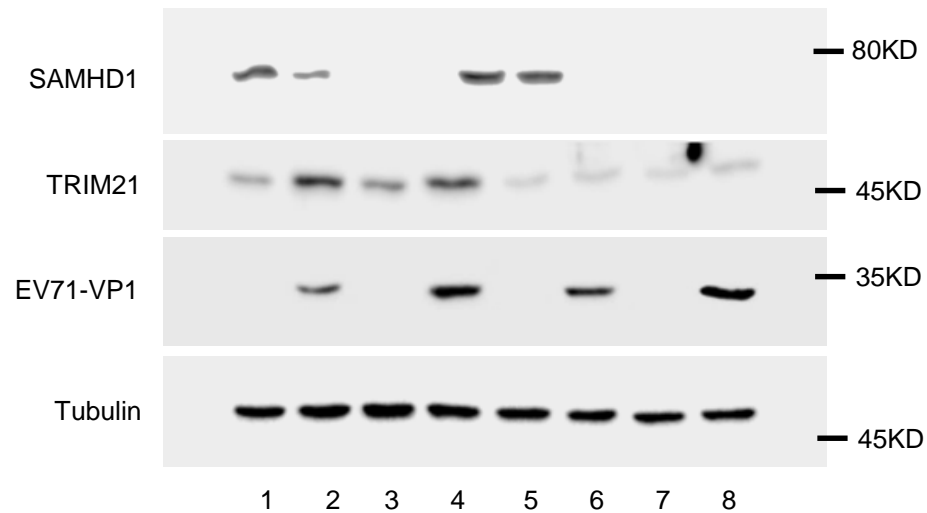

F

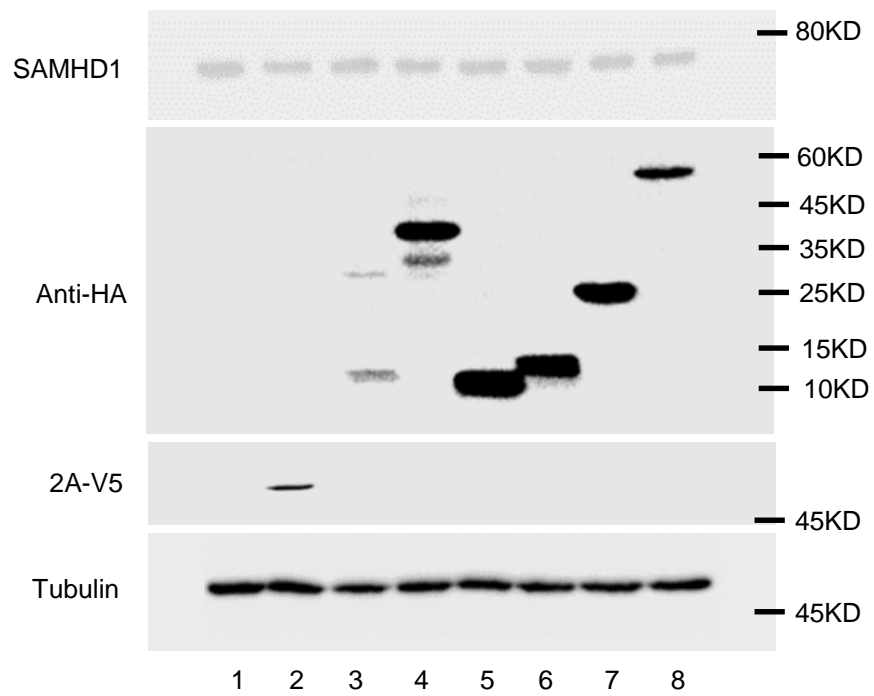

Fig. EV3 source data

Supplement: Supplementary file 3 — Source Data for Expanded View [file EMBR-21-e47528-s010.zip › Source_Data_for_EV_Figures/Fig_EV3/Source_Data_for_FigEV3.pdf]

A

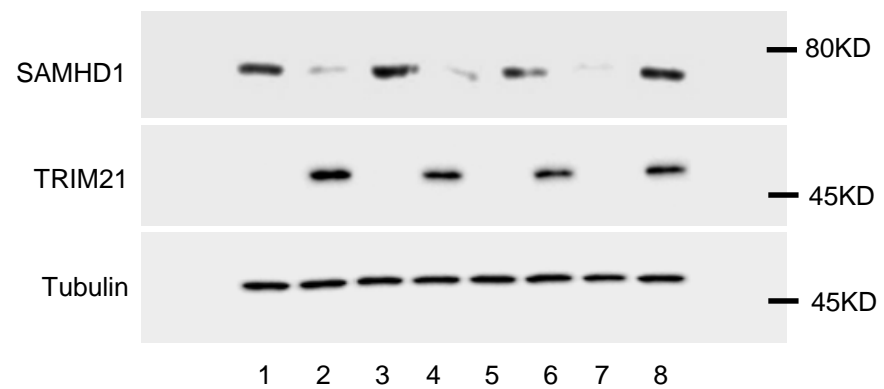

D

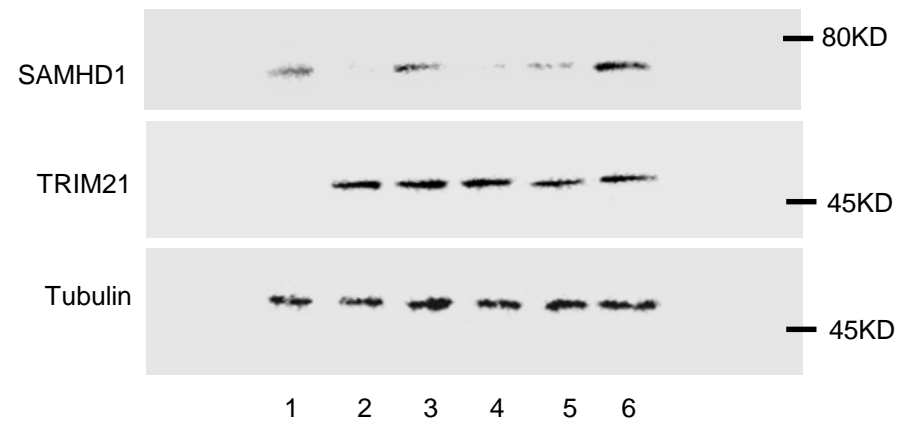

G

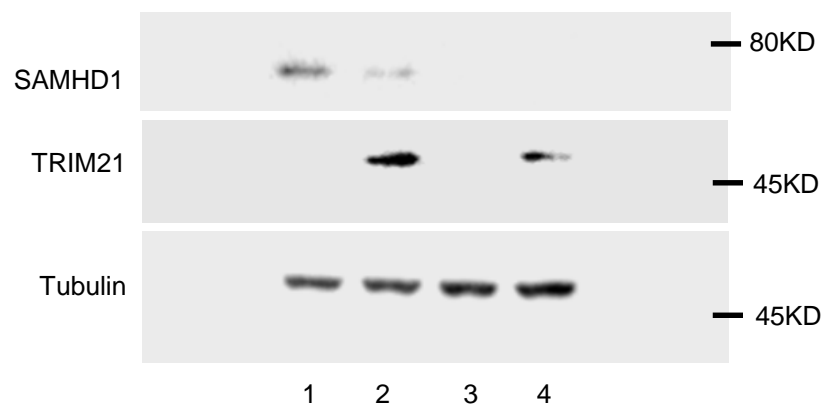

Fig. EV4 source data

Supplement: Supplementary file 3 — Source Data for Expanded View [file EMBR-21-e47528-s010.zip › Source_Data_for_EV_Figures/Fig_EV4/Source_Data_for_FigEV4.pdf]

A

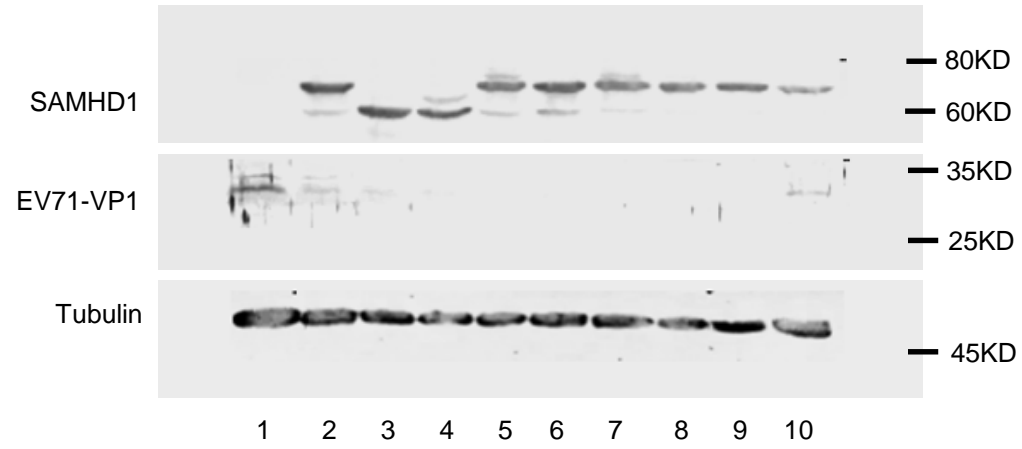

B

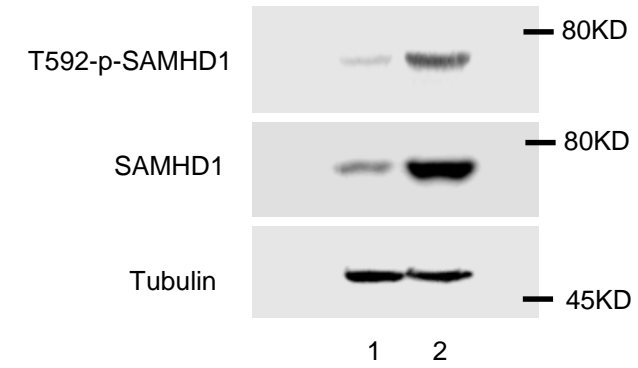

C

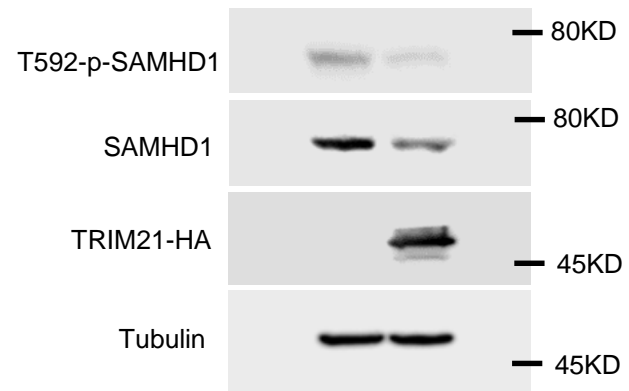

D

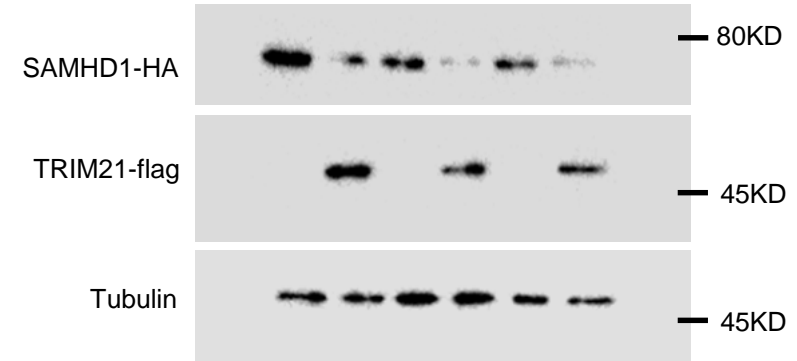

E

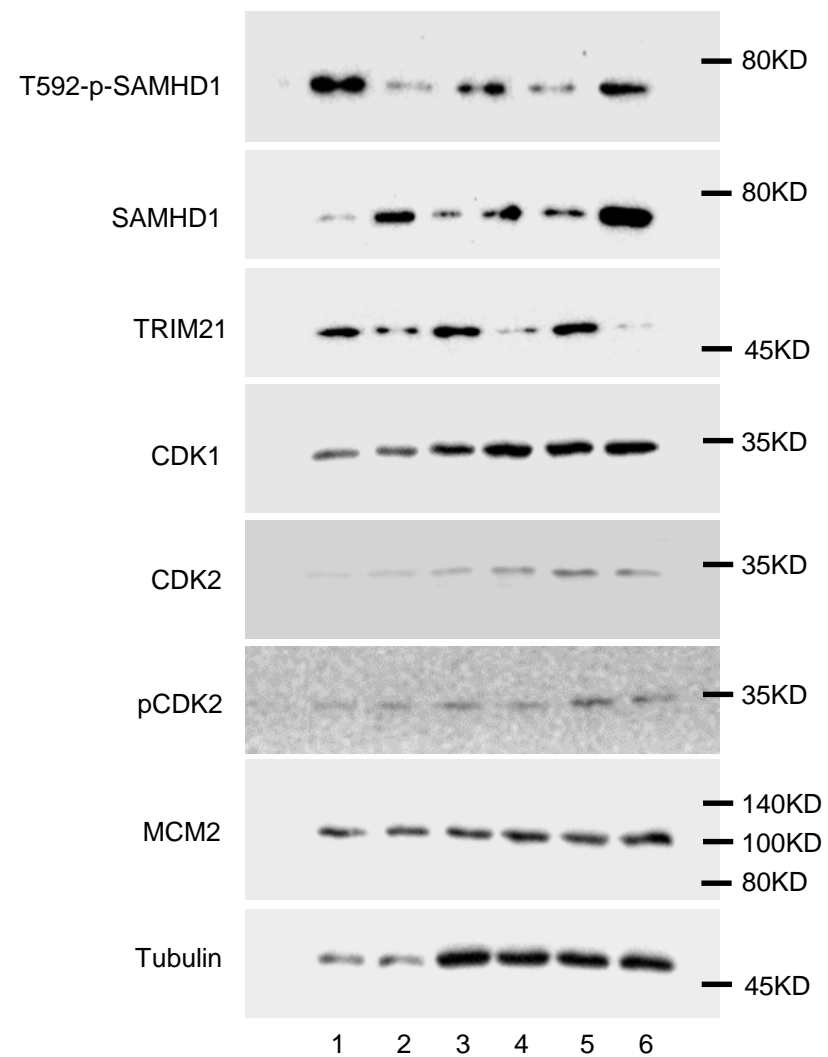

F

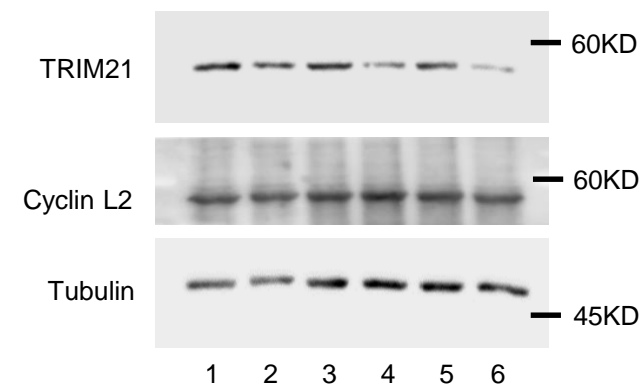

Fig. EV5 source data

Supplement: Supplementary file 3 — Source Data for Expanded View [file EMBR-21-e47528-s010.zip › Source_Data_for_EV_Figures/Source_Data_for_FigEV5.pdf]

A

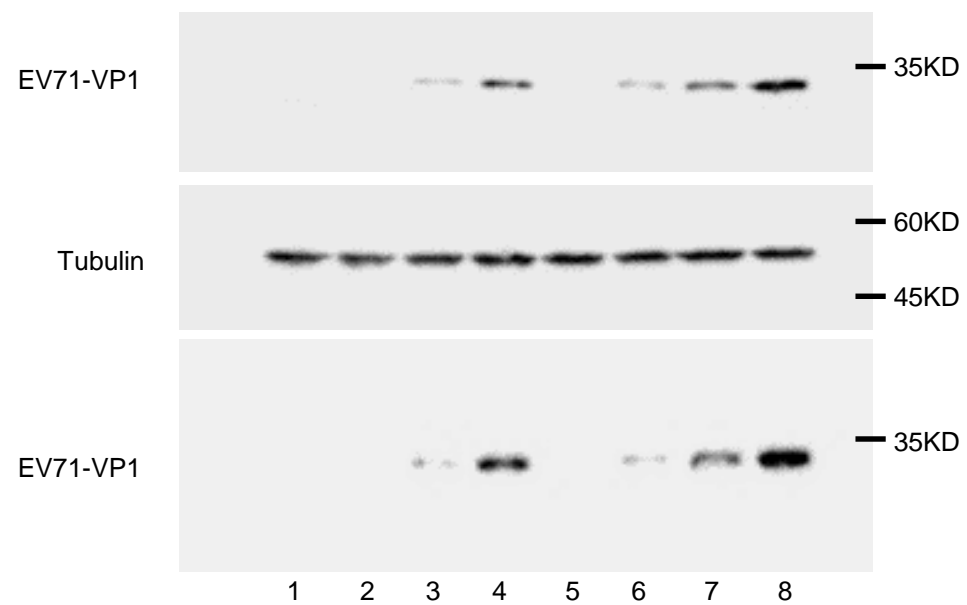

D

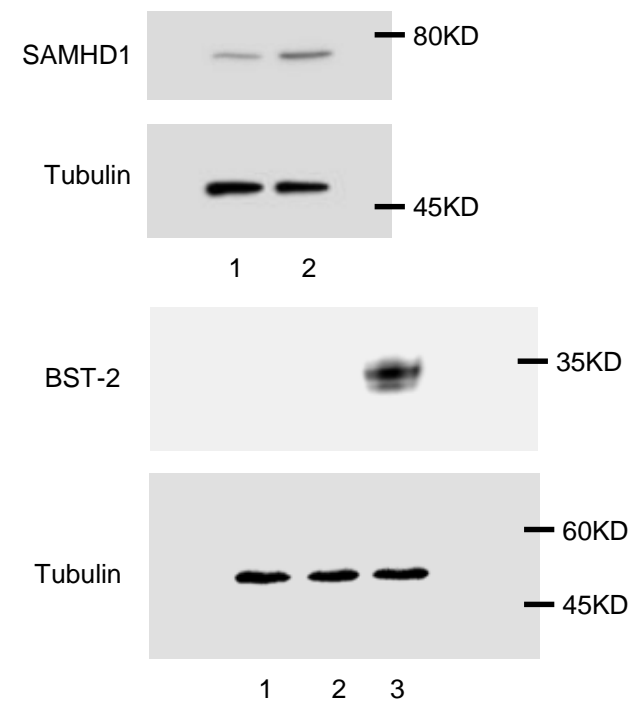

E

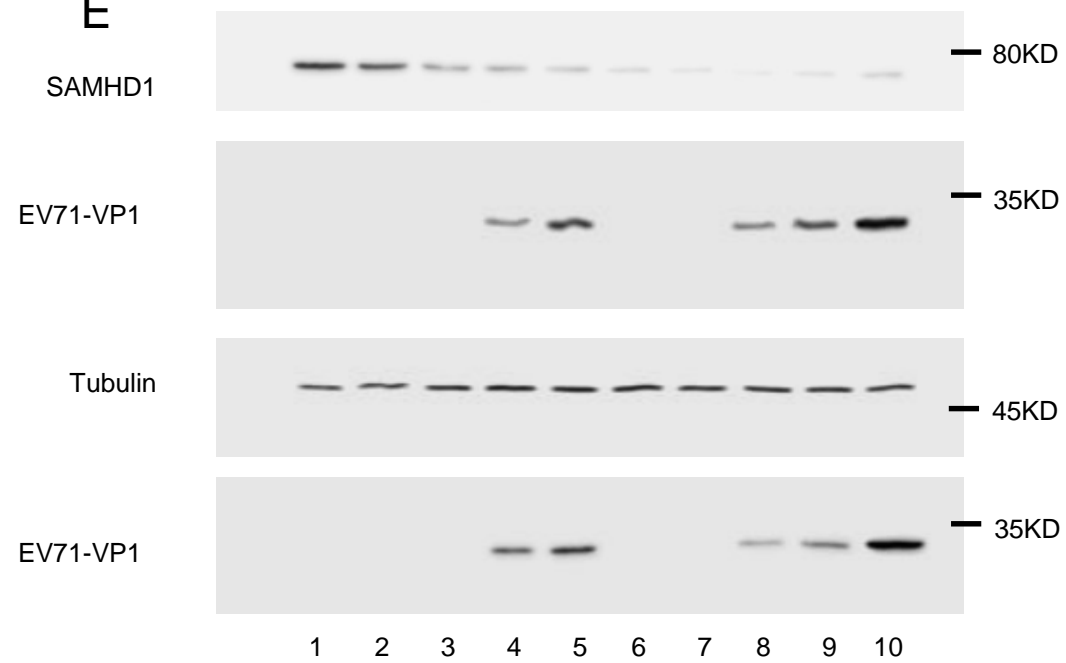

H

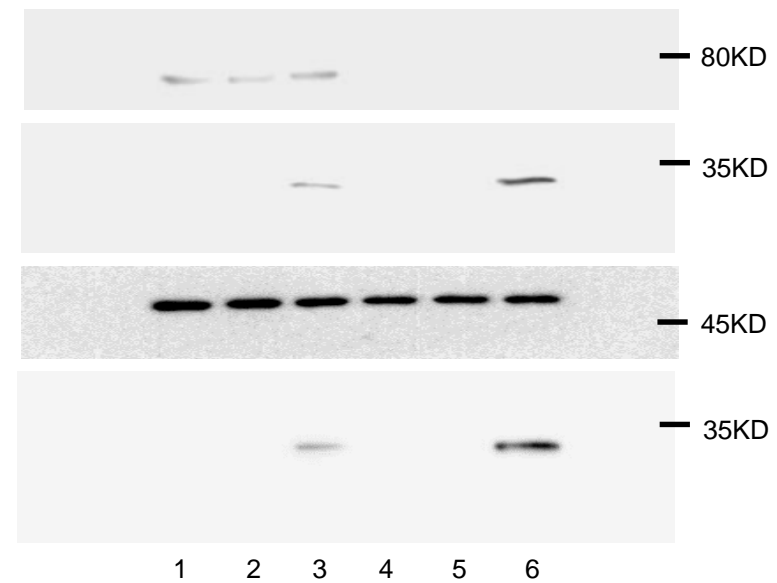

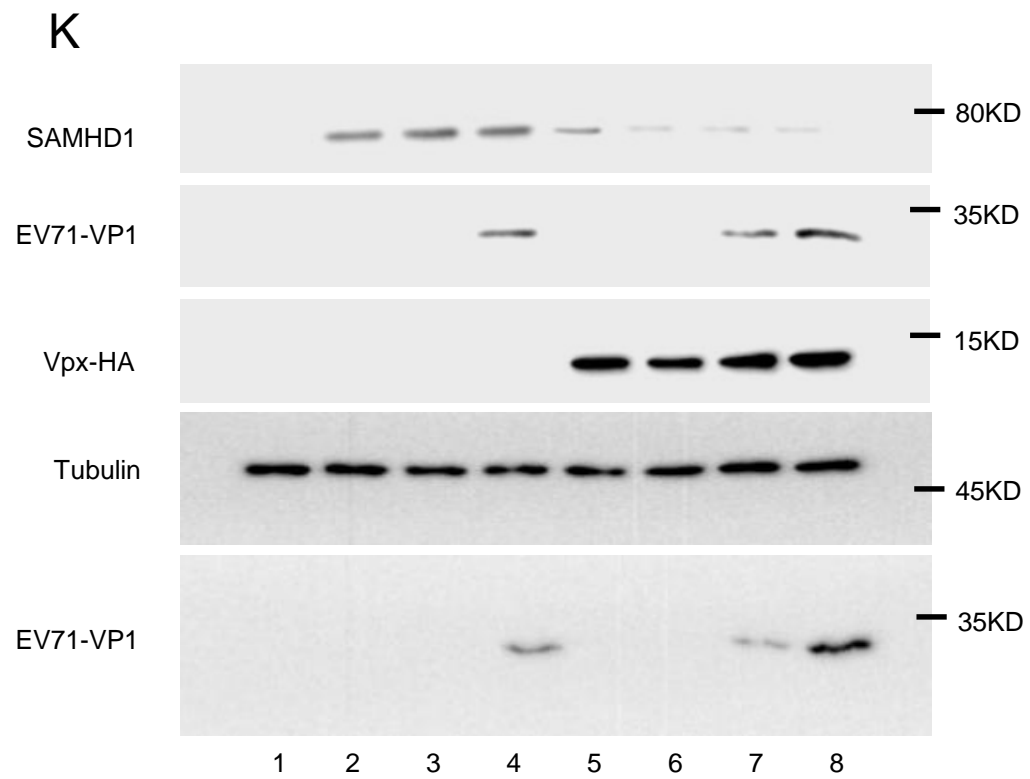

Fig. 1 source data

Supplement: Supplementary file 5 — Source Data for Figure 1 [file EMBR-21-e47528-s003.zip › Source_Data_for_Fig1/Source_Data_for_Fig1.pdf]

A

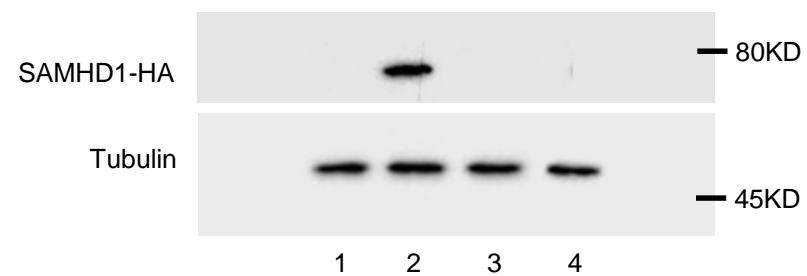

B

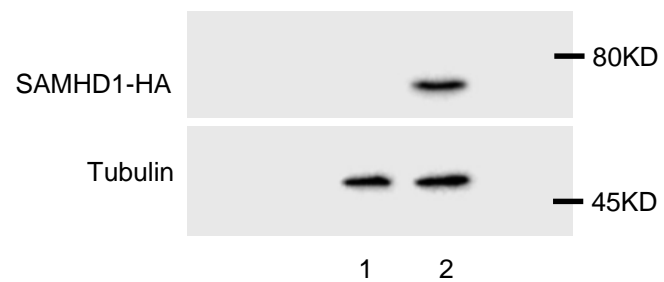

C

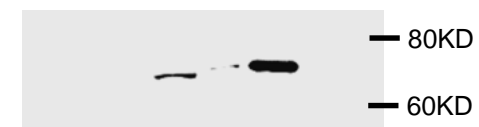

D

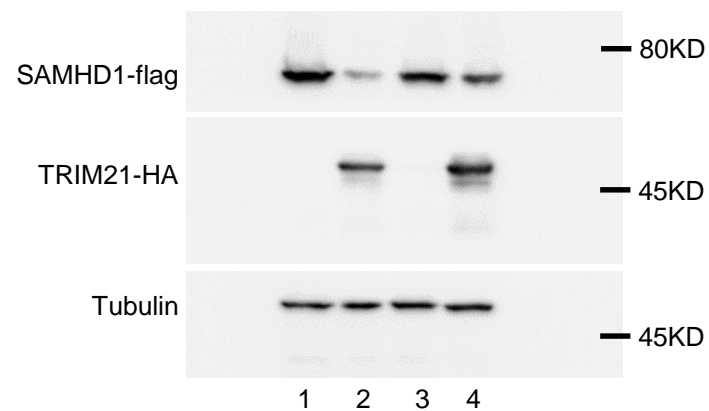

E

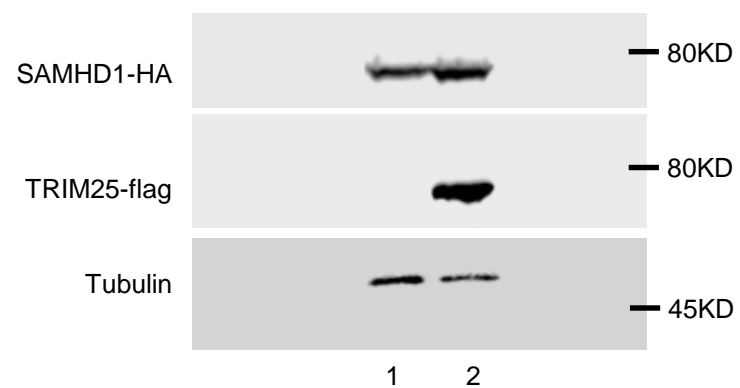

F

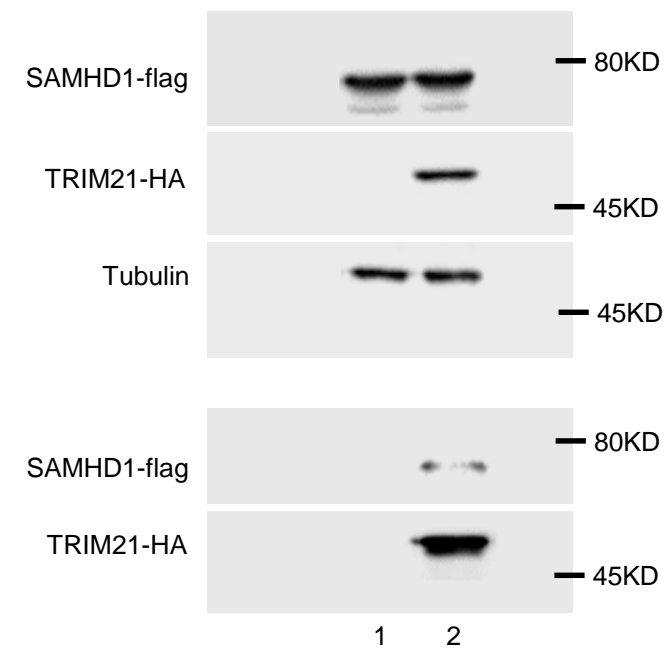

G

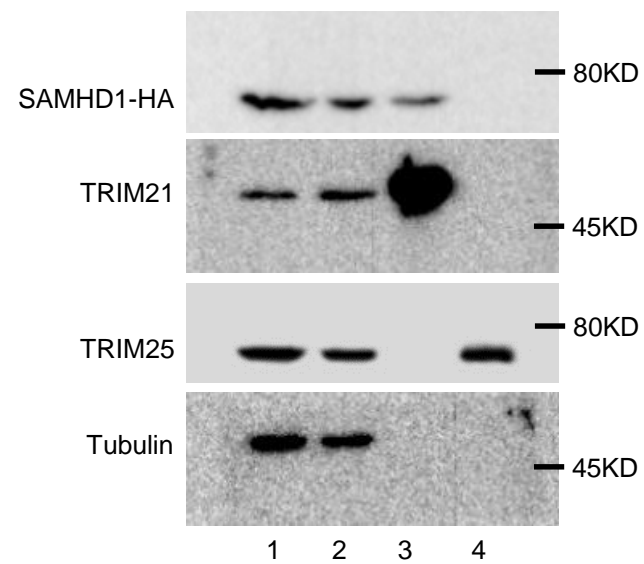

I

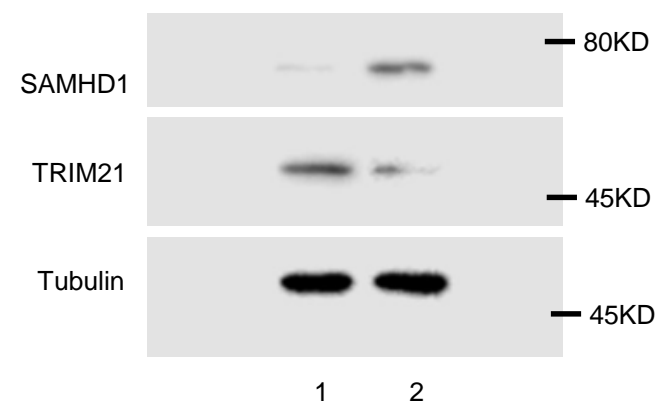

Fig. 2 source data

Supplement: Supplementary file 6 — Source Data for Figure 2 [file EMBR-21-e47528-s004.zip › Source_Data_for_FIg2/Source_Data_for_Fig2.pdf]

A

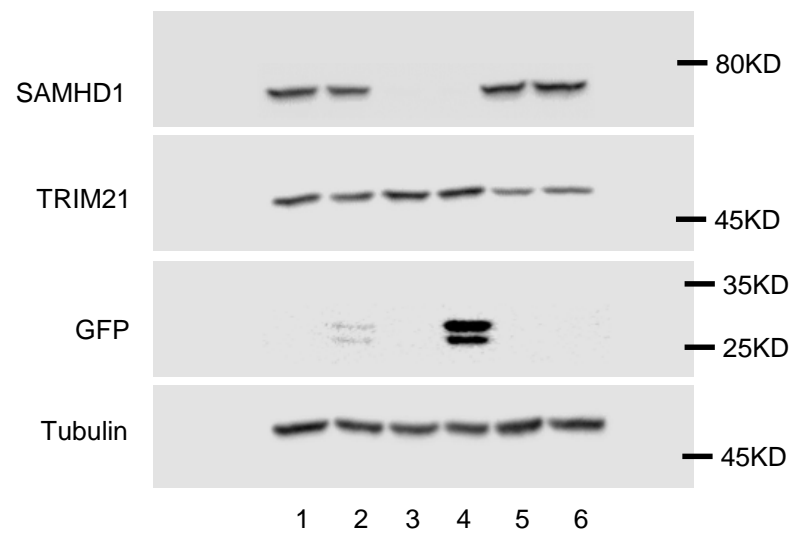

D

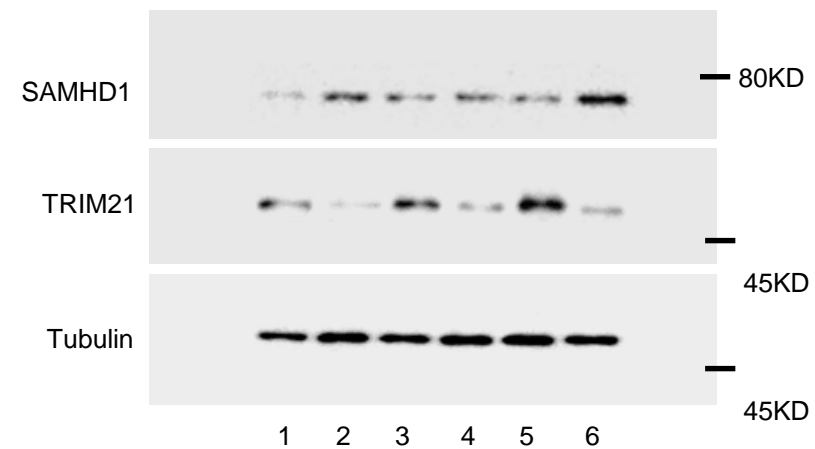

G

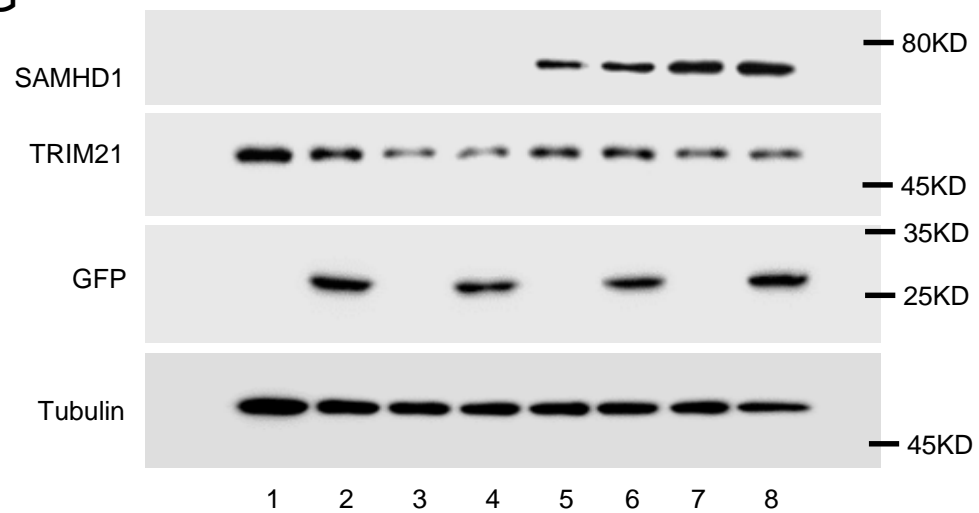

Fig. 5 source data

Supplement: Supplementary file 9 — Source Data for Figure 5 [file EMBR-21-e47528-s007.zip › Source_Data_for_Fig5/Source_Data_for_Fig5.pdf]

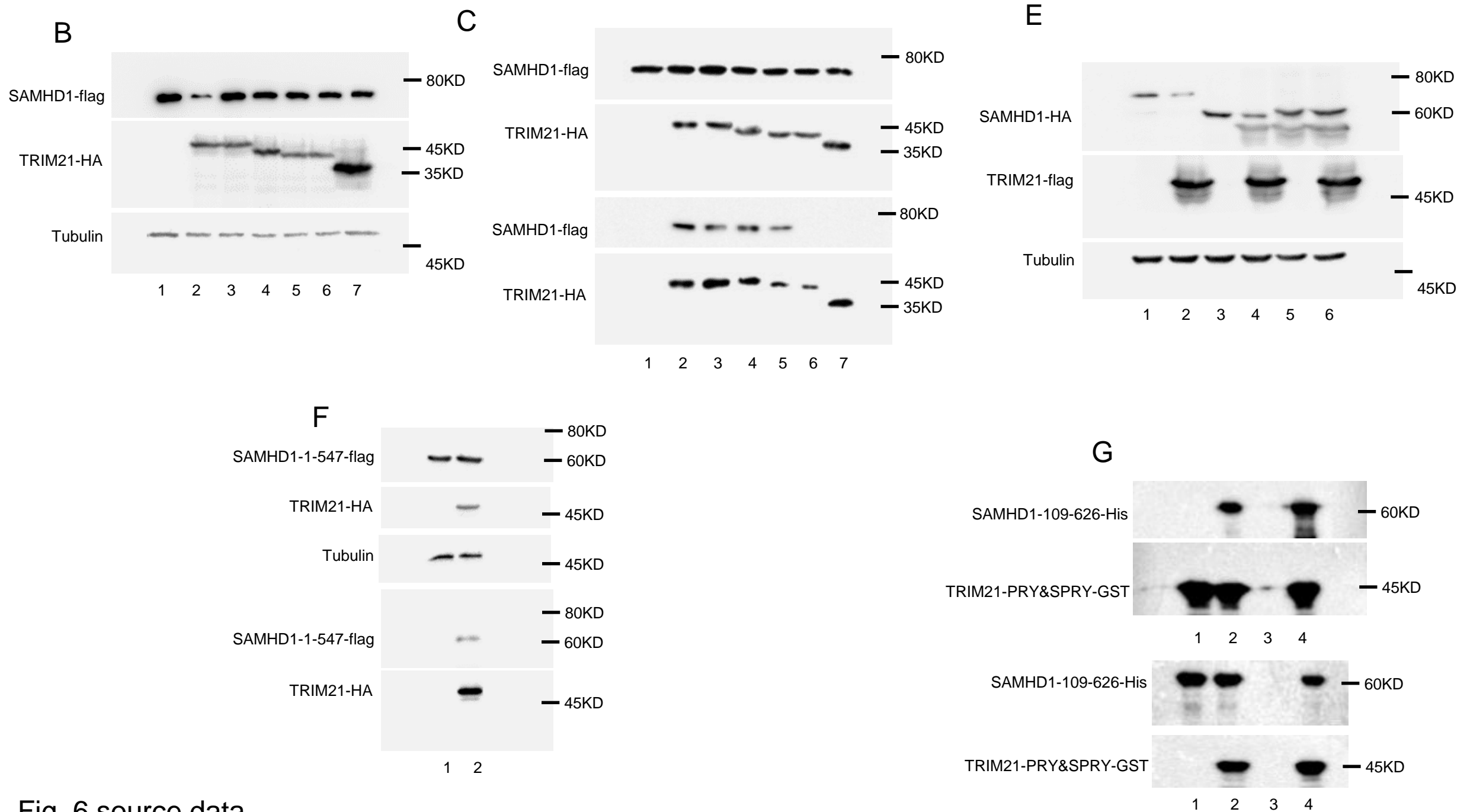

Fig. 6 source data

Supplement: Supplementary file 10 — Source Data for Figure 6 [file EMBR-21-e47528-s008.pdf]

A

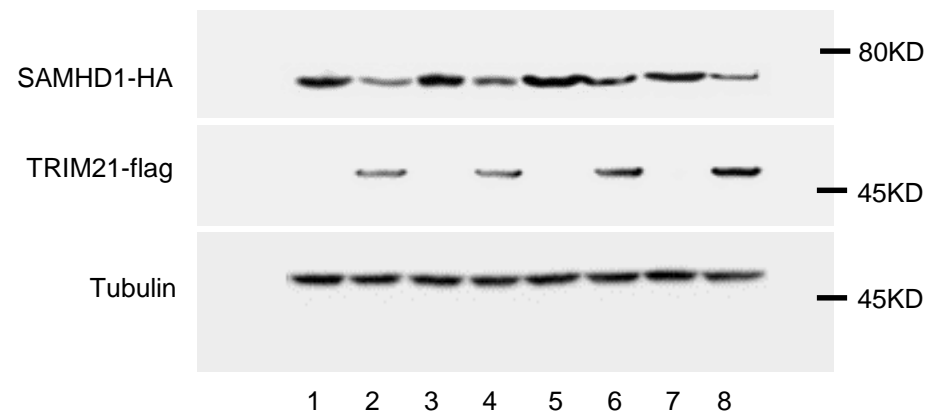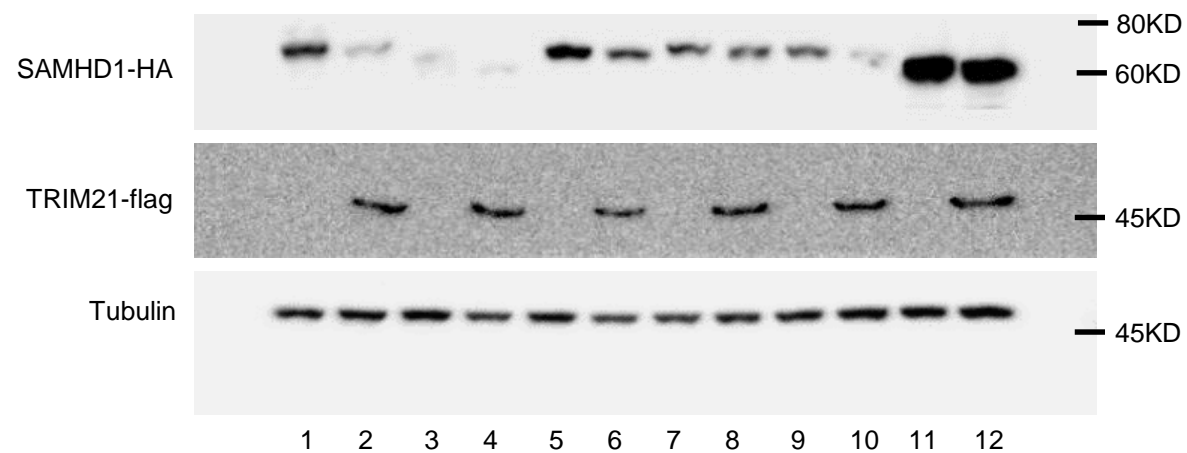

D

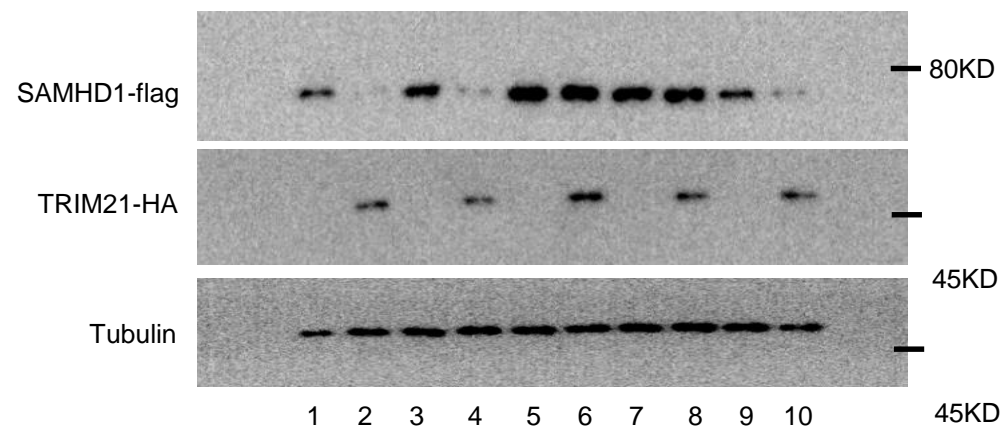

E

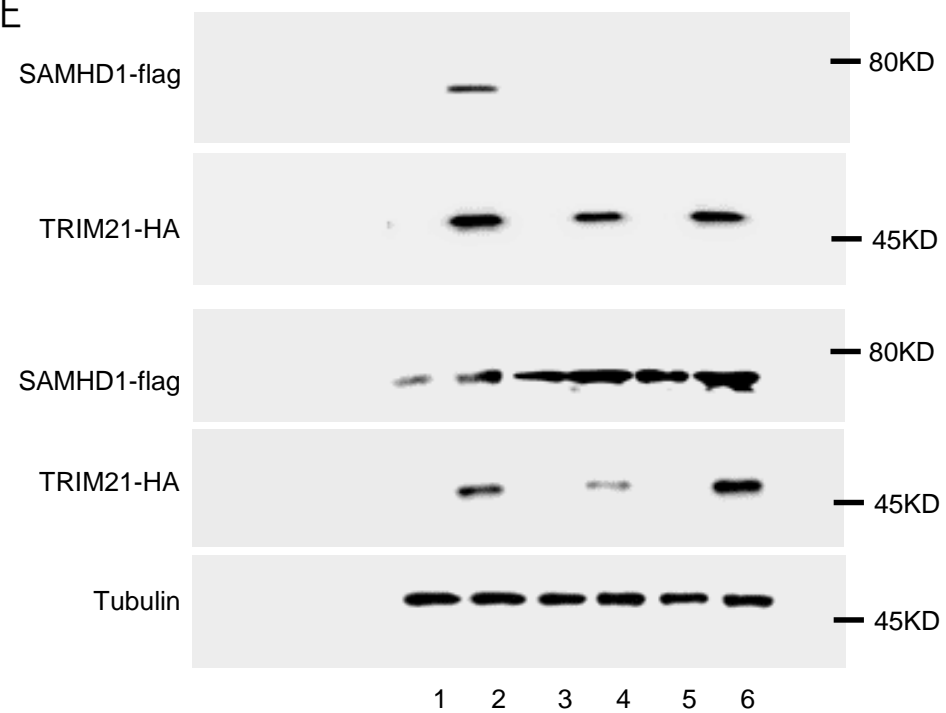

G

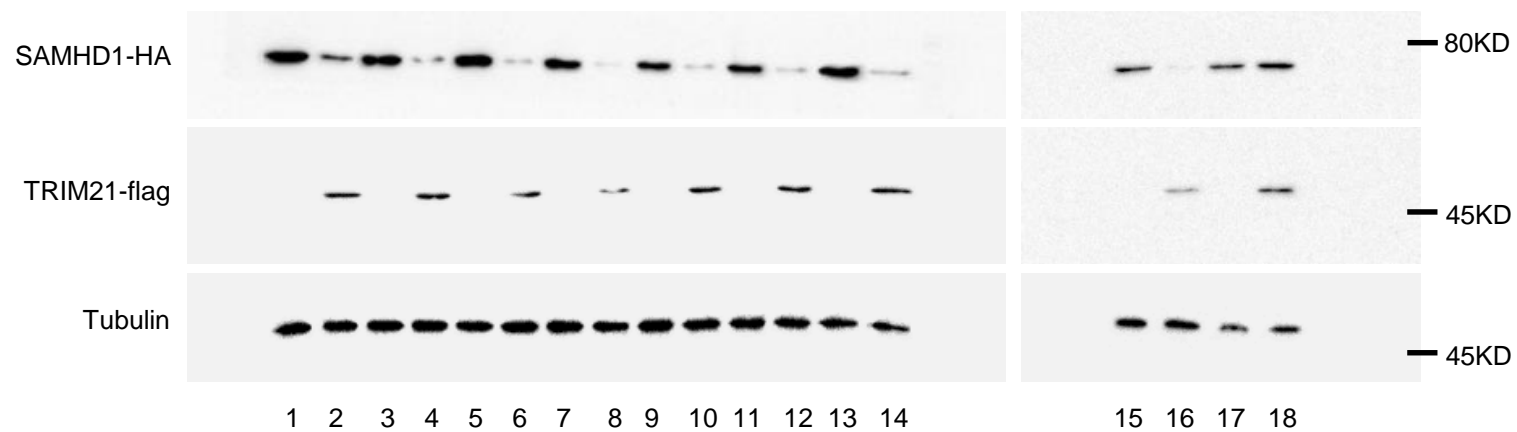

H

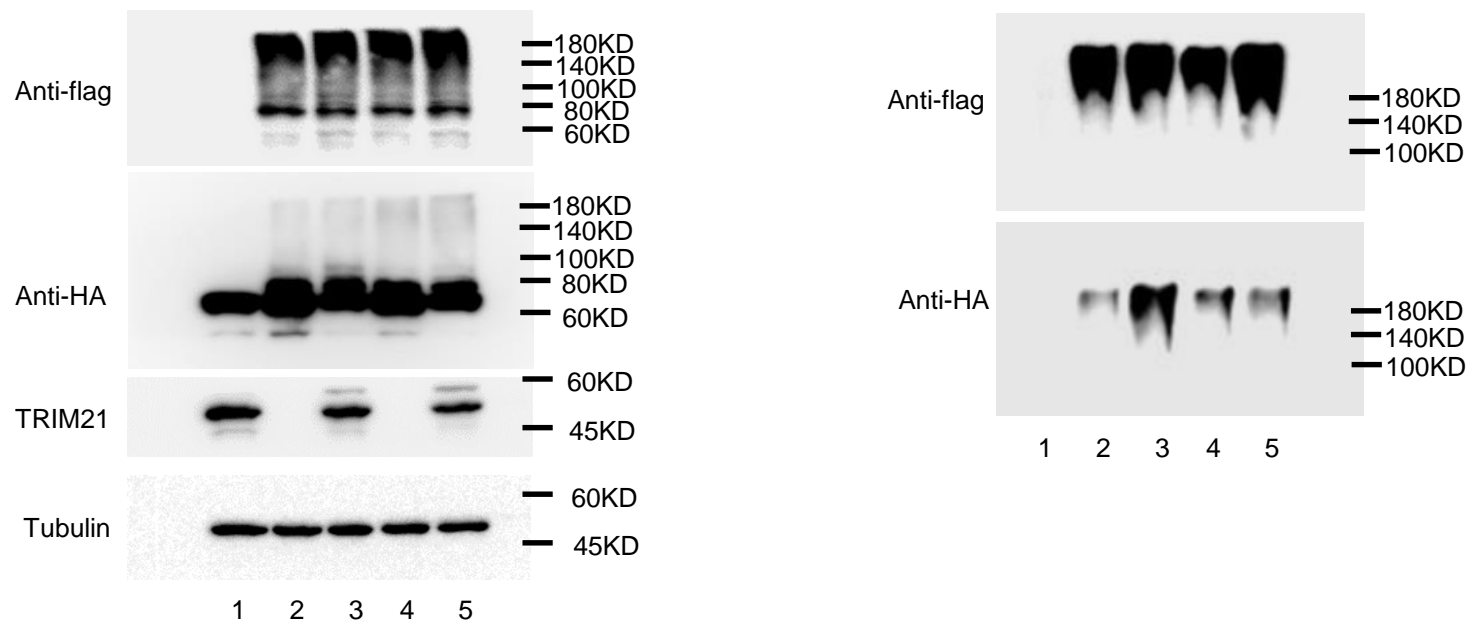

I

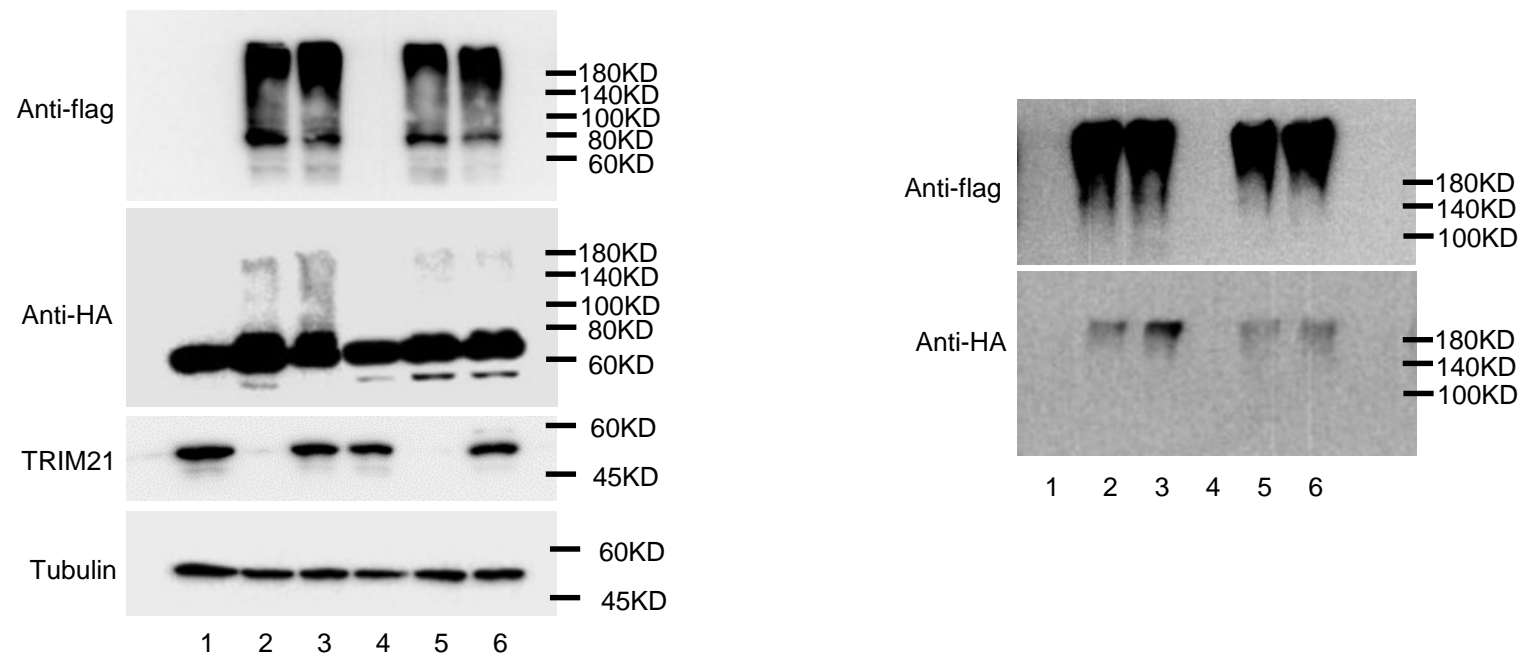

J

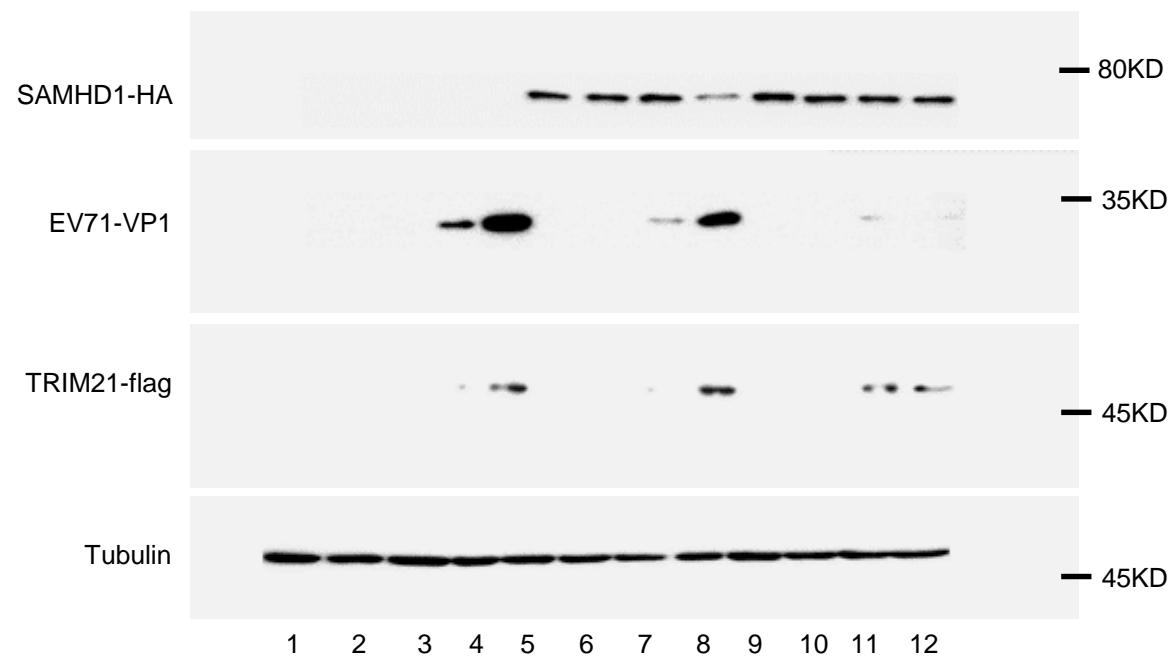

Fig. 7 source data

Supplement: Supplementary file 11 — Source Data for Figure 7 [file EMBR-21-e47528-s009.pdf]
