# Supplementary material for: TRIM21‐mediated proteasomal degradation of SAMHD1 regulates its antiviral activity
Source: EMBO Rep. 2019 Dec 4;21(1):e47528. doi: 10.15252/embr.201847528 (PMC6944907; doi:10.15252/embr.201847528)
Supplement: Supplementary file 7 — Source Data for Figure 3 [file EMBR-21-e47528-s005.zip › Source_data_for_Fig3/Source_Data_for_Fig3.pdf]

B

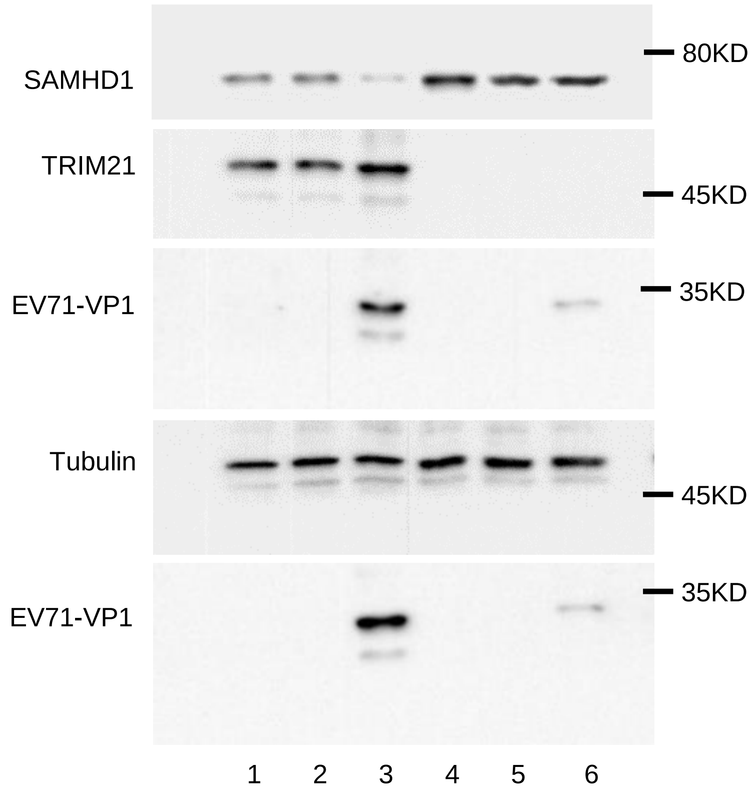
SAMHD1
TRIM21
EV71-VP1
Tubulin
EV71-VP1
80KD
45KD
35KD
45KD
35KD
1
2
3
4
5
6

E

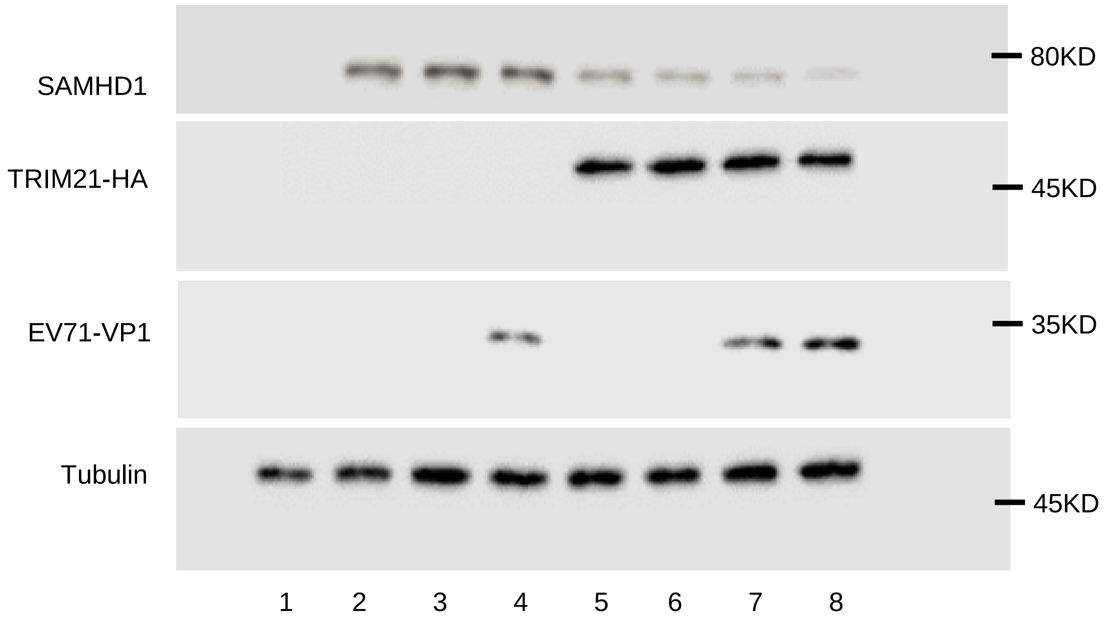
SAMHD1
TRIM21-HA
EV71-VP1
Tubulin
80KD
45KD
35KD
45KD
1
2
3
4
5
6
7
8

G

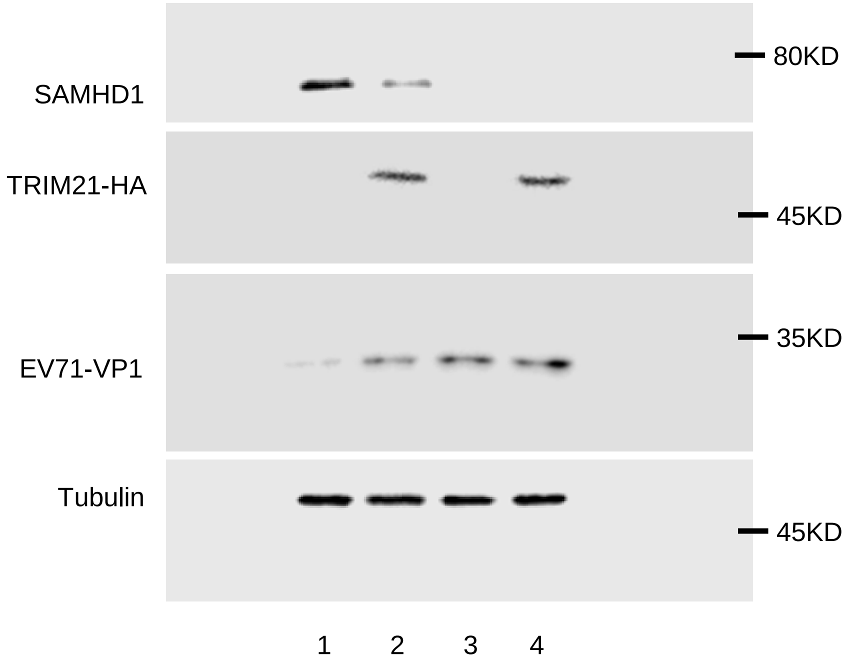

Fig. 3 source data
